# Multi-compartmental modeling of SORLA’s influence on amyloidogenic processing in Alzheimer’s disease

**DOI:** 10.1186/1752-0509-6-74

**Published:** 2012-06-22

**Authors:** Angelyn Lao, Vanessa Schmidt, Yvonne Schmitz, Thomas E Willnow, Olaf Wolkenhauer

**Affiliations:** 1 Department of Systems Biology and Bioinformatics, Institute of Computer Science, University of Rostock, Ulmenstrasse 69, Rostock, 18057, Germany; 2 Max-Delbrück-Center for Molecular Medicine, Robert-Roessle-Str. 10, Berlin, D-13125, Germany; 3 Stellenbosch Institute for Advanced Study (STIAS), Stellenbosch, 7600, South Africa

**Keywords:** Amyloidogenic processing, Compartmental modeling, LR11, Secretases, SORL1, VPS10P domain receptors

## Abstract

**Background:**

Proteolytic breakdown of the amyloid precursor protein (APP) by secretases is a complex cellular process that results in formation of neurotoxic Aβ peptides, causative of neurodegeneration in Alzheimer’s disease (AD). Processing involves monomeric and dimeric forms of APP that traffic through distinct cellular compartments where the various secretases reside. Amyloidogenic processing is also influenced by modifiers such as sorting receptor-related protein (SORLA), an inhibitor of APP breakdown and major AD risk factor.

**Results:**

In this study, we developed a multi-compartment model to simulate the complexity of APP processing in neurons and to accurately describe the effects of SORLA on these processes. Based on dose–response data, our study concludes that SORLA specifically impairs processing of APP dimers, the preferred secretase substrate. In addition, SORLA alters the dynamic behavior of β-secretase, the enzyme responsible for the initial step in the amyloidogenic processing cascade.

**Conclusions:**

Our multi-compartment model represents a major conceptual advance over single-compartment models previously used to simulate APP processing; and it identified APP dimers and β-secretase as the two distinct targets of the inhibitory action of SORLA in Alzheimer’s disease.

## Background

The amyloid precursor protein (APP) is a type-1 membrane protein expressed in neurons, which is closely linked to the etiology and pathology of Alzheimer’s disease (AD) [1]. APP undergoes two mutually exclusive processing pathways resulting in the formation of multiple soluble and membrane-associated fragments from this precursor polypeptide. Of particular relevance to AD is the amyloidogenic pathway whereby APP is first cleaved by β-secretase and subsequently by γ-secretase to produce the amyloid-β peptide (Aβ), a 40 to 42 amino acid fragment derived from part of the extracellular and the transmembrane domains of APP. According to the amyloid hypothesis, neurotoxic oligomers and senile plaques formed by Aβ cause neuronal dysfunction and cell loss in AD [2,3]. In the alternative pathway, APP is first cleaved by α-secretase, instead of β-secretase, resulting in the destruction of the Aβ peptide sequence in APP (non-amyloidogenic pathway). Adding to the complexity of APP processing is the distinct trafficking route of the precursor through intracellular compartments where the various secretases reside [4,5]. Thus, newly synthesized APP molecules move through the constitutive secretory pathway from the trans-Golgi network (TGN) to the cell surface where most are subjected to non-amyloidogenic processing by α-secretase. However, approximately 10% of the precursors remain intact and internalize from the cell surface into endosomal compartments where amyloidogenic processing is initiated by β-secretase cleavage.

In recent years, much attention has been focused on the analysis of factors that influence APP processing and that may contribute to the elevated Aβ levels seen in patients with AD. One such modifier is SORLA, a 250-kDa type-1 membrane glycoprotein widely expressed in neurons in the brain [6,7]. It is a member of a family of mammalian proteins that share a structural similarity with the vacuolar protein sorting 10 protein (VPS10p), a y*east* sorting receptor that transports carboxypeptidase Y from the TGN to the vacuole [8]. SORLA is proposed to act as a retention factor for APP in the TGN, preventing the release of precursor molecules into the processing pathways. Consequently, over-expression of SORLA in neurons prevents the targeting of APP from TGN to the cell surface and to endosomes and reduces the production of Aβ peptides [9-11]. The importance of SORLA for AD is further supported by low levels of receptor expression seen in patients suffering from the disease [12,13].

In a first approach to simulate amyloidogenic processing in AD, Schmidt and colleagues [14] developed a single-compartment model to describe APP processing. While this model had been valuable to establish the kinetics of amyloidogenic processing and the quantitative contribution of SORLA to this pathway, this single-compartment model fell short of accurately describing the complexity of APP processing in cells. It remained unclear to what extent SORLA may affect APP monomer versus dimer processing and in what compartment of the cell its activity may be most relevant. Also, a possible influence of SORLA on the dynamics of β-secretase remained unclear. Such an effect had been postulated previously based on studies in cultured cells [15].

To answer these questions, we established a multi-compartment model that represents APP processing in both its monomeric and dimeric forms. The formalism of this model is developed to integrate experimental evidences from previous biochemical and cell biological studies [9-11,13,15-17]. We combined our multi-compartment model with the recent dose–response data of APP and soluble APP products by Schmidt and colleagues [14]. The data were used to estimate the parameter values of our model. Using our multi-compartment model, we (i) established the activity distribution of APP in various compartments, and (ii) traced the activity distribution of APP, α-secretase, β-secretase and SORLA in the monomeric and dimeric processing of APP. Our simulation results showed that the decrease in total APP processing is primarily due to the influence of SORLA on APP dimer processing. Moreover, the simulations of our multi-compartment model demonstrated how SORLA alters the dynamical behavior of β-secretase, providing new insights into the mechanism of action of this important AD risk factor.

## Results and discussion

### Multi-compartmental modeling of APP processing in the presence or absence of SORLA

Probably more than any other major disease entity, AD is a pathological processes influenced by subtle quantitative changes in protein concentration and activity. Thus, common approaches in experimental AD research, using protein overexpression or gene-inactivation, are inadequate to study the effects of incremental changes in target protein levels on risk of neurodegeneration.

In our previous study [14], we have undertaken the first attempt to approach risk factors in AD through quantitative modeling. To do so, we have simulated the quantitative contribution of SORLA to proteolytic processing of APP, a central pathway in AD. We have chosen SORLA as a target for simulation because it represents one of the major genetic risk factors in AD. More importantly, solid experimental evidence had established the molecular mechanism of SORLA action, acting as an intracellular sorting receptor for APP that prevents proteolytic breakdown of the precursor protein into neurotoxic Aβ peptides. In [14], we have been able mathematically confirm hypotheses, derived from prior experimental work. In particular, we have confirmed the strict linear relationship between SORLA concentrations and efficiency of APP processing, and we have uncovered the ability of SORLA to prevent dimerization of APP, preventing the formation of high-affinity substrates for secretases.

While our initial study has been met with great enthusiasm in the field, it clearly falls short of addressing major aspects of SORLA activity in the cell biology of AD. Thus, for sake of simplification, our earlier study assumed a single-compartment model for simulation of the affects of SORLA levels on APP processing rates. Accordingly, it ignores the fact that APP follows a complex intracellular trafficking pathway whereby this protein moves between the TGN, cell surface, and endosomes where the various interacting proteins reside. In fact, it has the ability to show how SORLA affects APP transport between various cell compartments in neurons that initially sparked interest in this protein.

**Figure 1:**
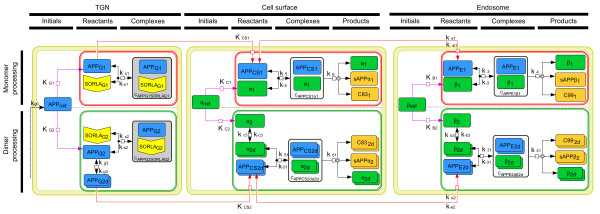
**Biochemical network of a multi-compartmental model describing the influence of SORLA in APP processing.** The three main yellow compartments in the network are the trans-Golgi network (TGN), the cell surface and the endosomes. Each compartment is subdivided into two subcompartments: a red subcompartment for APP monomer processing and a green one for dimer processing. The monomeric forms of APP, SORLA, α-secretase, and β-secretase, within the two subcompartments, are annotated differently: *APP*_*G1*_, *SORLA*_*G1*_, *α*_*1*_, and *β*_*1*_ for monomer processing, and *APP*_*G2*_, *SORLA*_*G2*_, *α*_*2*_, and *β*_*2*_ for dimer processing. Moreover, *APP*_*G2*_, *α*_*2*_, and *β*_*2*_ undergo dimerization before the start of APP processing. In the TGN, *SORLA*_*G1*_ binds to *APP*_*G1*_ in red subcompartment while *SORLA*_*G2*_ to *APP*_*G2*_ in the green subcompartment. At the cell surface, *APP*_*CS1*_ and *APP*_*CS2d*_ are cleaved by *α*_*1*_ and *α*_*2d*_ producing soluble fragments encompassing the extracellular domain of APP. These fragments are called soluble (s) *sAPPα*_*1*_ and *sAPPα*_*2*_, respectively. In addition, α-secretase cleavage produces a membrane-associated fragment containing the membrane anchor and the cytoplasmic tail, denoted C83. In the endosomes, APP molecules that escaped cleavage by α-secretase (*APP*_*E1*_ and *APP*_*E2d*_) are cleaved by *β*_*1*_ and *β*_*2d*_. Cleavage results in production of the soluble fragments of the extracellular APP domain (*sAPPβ*_*1*_ and *sAPPβ*_*2*_) and in the membrane-tethered fragments *C99*_*1*_ and *C99*_*2d*_. C99 includes the Aβ peptide sequence and represents the substrate for γ-secretase cleavage. The diagram was produced with Cell Designer 4.0 [18,19].

For the present work, a single-compartment model, describing the influence of SORLA in APP processing [14], was extended into a multi-compartmental model. The extended model addresses the important aspect of the cell biology of SORLA by assuming a three-compartment model that is based on experimental data. The biochemical network illustrating this multi-compartmental model can be found in Figure 1. The notation that is used in this network is described in Additional file 1: Table S1.

It is likely that there are many other proteins contribute to the processing of APP and the generation of neurotoxic Aβ peptides. However, unlike many proposed AD risk factors, the mechanism of action for SORLA has been established in numerous studies in cell cultures, in animal models, and even in patients providing a solid base for theoretical simulations. In particular, we have specifically addressed the caveat that this model focuses on pathways related to SORLA action, and that further studies will be required to sequentially add more risk factors to this model. Such approaches will require a profound understanding of the function of such risk factors; - an endeavor that clearly exceeds the scope of the present manuscript.

The choice of the compartments considered in Figure 1 was based on the different locations where APP was shown to interact with SORLA, with α-, and with β-secretases. The corresponding three compartments are the TGN, the cell surface and the endosomes [9,15,20], respectively. Note that the transport of APP among these compartments indirectly interconnects these three compartments to one another. As SORLA affects the initial cleavage of APP by α- and β-secretases [11], the rate limiting steps that determine the extent of amyloidogenic processing, further processing steps involving γ-secretase were not included in this model.

In order to accommodate the monomeric and dimeric forms of APP, each compartment was further divided into two subcompartments (Figure 1): a “red” subcompartment for APP monomer processing and a “green” one for APP dimer processing. Notice that the monomeric forms of APP, SORLA, α-secretase, and β-secretase, within the two subcompartments, were annotated differently: *APP*_*G1*_, *SORLA*_*G1*_, *α*_*1*_, and *β*_*1*_ for monomer processing, and *APP*_*G2*_, *SORLA*_*G2*_, *α*_*2*_, and *β*_*2*_ for dimer processing. Even so, the components from the two subcompartments were linked to each other via *APP*_*init*_, *α*_*init*_, and *β*_*init*_. Moreover, *APP*_*G2*_, *α*_*2*_, and *β*_*2*_ undergo dimerization before the beginning of APP dimer processing. That is, two *APP*_*G2*_, *α*_*2*_, and *β*_*2*_ monomers dimerize in order to give their corresponding dimeric forms. Conversely, these dimers can dissociate to generate their respective monomers. Note that subscript ‘1’ was assigned to the reactants and products in monomer processing while subscript ‘2’ for those in dimer processing. In addition, we used subscripts ‘G’, ‘CS’, and ‘E’ for APP in TGN, at the cell surface and in the endosomes, respectively.

Up to this point, we had described the different forms of APP, α-secretase, and β-secretase in the diverse compartments, prior to the beginning of APP processing. Because SORLA interacts with APP in a 1:1 stochiometric complex [9,16], the model described how SORLA strictly interacts with APP-monomers (but not dimers) to form an APP-SORLA complex. Consequently, this interaction is responsible for the diminished amount of APP-monomers (*APP*_*G1*_) and APP-dimers (*APP*_*G2d*_) transported from the TGN to the cell surface. This interaction decreases the amount of APP-monomers (*APP*_*CS1*_) and APP-dimers (*APP*_*CS2d*_) ending up in the endosomes as *APP*_*E1*_ and *APP*_*E2d*_. Moreover, in order to determine whether SORLA will have a similar influence on the monomer and dimer processing, the binding affinity assigned to *APP*_*G1*_*SORLA*_*G1*_ in monomer processing is different to that of *APP*_*G2*_*SORLA*_*G2*_ in dimer processing.

After the interaction of SORLA and APP in the TGN, the remaining *APP*_*G1*_ and *APP*_*G2d*_ are transported to the cell surface where APP processing begins within the non-amyloidogenic pathway. Then, a small part of *APP*_*CS1*_ and *APP*_*CS2d*_, which is not cleaved by α-secretase, are further transported from the cell surface to the endosomes, where the amyloidogenic pathway takes over. Notably, the interaction of APP and α-secretase at the cell surface leads to the formation of non-amyloidogenic products like sAPPα and C83; whereas the interaction of APP and β-secretase in the endosomes yields to the amyloidogenic products such as sAPPβ and C99. Our model was established in such a way that the dimeric form of secretases act only on the dimeric form of APP and the monomeric form of secretases act only on the monomeric form of APP.

The biochemical network (Figure 1) that we established, was translated into a system of ordinary differential equations (ODEs), describing temporal changes of molecular numbers for the network components as a function of interaction and cleavage processes. The model equations, their reduction, and a series of steps involved in model simulations are presented in the Materials and Methods section.

### Decrease in total amounts of sAPP products is mainly due to the influence of SORLA in dimer processing

**Figure 2:**
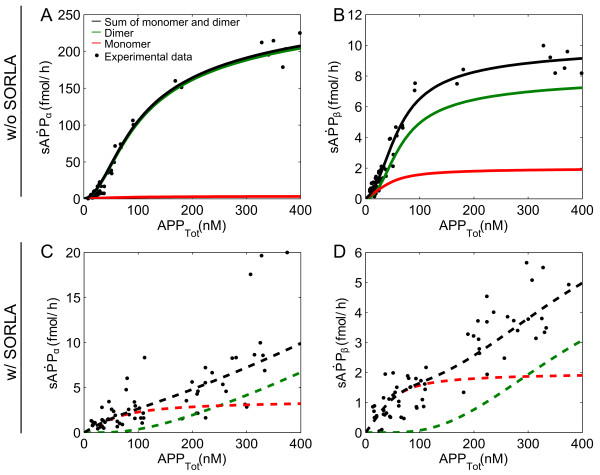
**Simulation results of the sAPPα and sAPPβ end products.** Simulation results of our multi-compartmental model (lines) for the various APP products are shown together with the actual data points obtained in biochemical experiments by Schmidt and colleagues [14]. The total amount of products (black line) is the sum of the products produced in monomer (red line) and in dimer processing (green line) pathways. In the absence of SORLA, the products produced in the dimer processing pathways more closely resemble the total amount of sAPPα (**A**) and sAPPβ (**B**). With SORLA, the amounts of sAPPα and sAPPβ that are produced in dimer processing are significantly reduced as compared to those in monomer processing (**C, D**).

With the multi-compartmental model, we showed in Figure 2 the corresponding model simulations for various APP products, namely, the products produced in monomer, in dimer, and in both processing pathways. The simulations of the parameterized mathematical model are in good agreement with recent experimental data by Schmidt and colleagues [14] (Figure 2A-D).

In the absence of SORLA, the sigmoidal curve that is characteristic for products produced in dimer processing (green lines in Figure 2A and B) has a strong impact on the sum of the products produced in monomer and in dimer processing pathways (black lines in Figure 2A and B). As such, it very well describes the experimental data sets for sAPPα and sAPPβ (black dots in Figure 2A and B, respectively).

**Figure 3:**
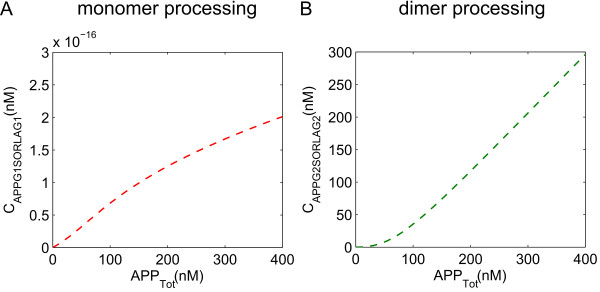
**Complex formation of APP-SORLA in monomer and in dimer processing.** Simulations of the influence of SORLA on APP processing on the complex formation of APP-SORLA in monomer (**A**) and in dimer (**B**) processing are shown. There is more APP bound to SORLA in dimer processing (**B**) than in monomer processing (**A**).

Surprisingly, in the presence of SORLA, one observes from the simulations a significant decrease in the products produced in dimer processing (green lines in Figure 2C and D) as compared to those in monomer processing (red lines in Figure 2C and D). In particular, the analysis showed that at a high level of SORLA activity (i.e. 100% of SORLA_Tot_ where SORLA_Tot_ equals 2.43 x 10^5^ fmol), there is obviously more APP bound to SORLA in dimer processing (Figure 3B) than in monomer processing (Figure 3A).

Taken together, our simulations shown in Figure 2 and Figure 3, strongly supported the hypothesis whereby SORLA prevents oligomerization of APP, thereby having a bigger impact on the products produced in dimer processing than in monomer processing.

### Intermediate levels of SORLA

**Figure 4:**
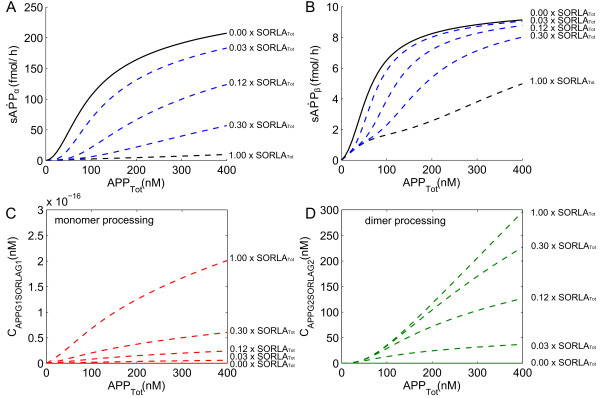
**APP processing at intermediate levels of SORLA.** Simulations of the influence of intermediate levels of SORLA on APP processing into the total amount of sAPPα (**A**) and total amount of sAPPβ (**B**), and on the complex formation of APP-SORLA in monomer (**C**) and in dimer (**D**) processing are shown. They are simulated in different intermediate levels of SORLA: from without SORLA, to 3%, 12%, 30%, and 100% of SORLA_Tot_ (where SORLA_Tot_ = 2.43 x 10^5^ fmol).

**Figure 5:**
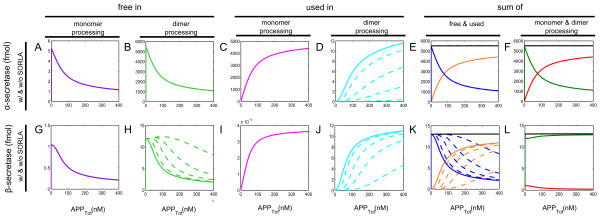
**Concentration values of the secretases at intermediate levels of SORLA.** Simulations of the influence of intermediate levels of SORLA on APP processing on the amount of α-secretase (**A-F**) and β-secretase (**G-L**) concentration. The term “used” refers to the complex formation of the secretases and APP, while the term “free” refers to the secretases that are not bound in a complex. There are five intermediate levels of SORLA, namely, 0% (solid line), 3%, 12%, 30%, and 100% (dashed line) of SORLA_Tot_ (where SORLA_Tot_ = 2.43 x 10^5^ fmol). When there is only solid line in a plot, it is because solid and dashed lines are superimposed. Starting from the first column, there shows the amount of α- (**A**) and β-secretase (**G**) that is free in monomer processing. In the second column, it shows the amount of α- (**B**) and β-secretase (**H**) that is free in dimer processing. The amount of α- (**C**) and β-secretase (**I**) used in monomer processing are shown in the third column, whereas those used in dimer processing (**D**, **J**) are shown in the fourth column. In the fifth column, there shows the total amount of α- (**E**) and β-secretase (**K**) that is free (blue line) and used (orange line) in the system. Lastly, there is the total amount of α- (**F**) and β-secretase (**L**) in monomer (blue line) and in dimer (orange line) processing of the system. The black lines in (**E**, **F**) and in (**K**, **L**) are the estimated total amount of α-and β-secretase, respectively. In particular, the black line in (**E**, **K**) represents the sum of the secretase concentration depicted by the blue and orange lines, while the one in (**F**, **L**) indicates the sum of the secretase concentration depicted by the red and green lines. Notice that the solid and dashed lines for both blue and orange colors deviates in (**K**). This, however, is not the case in (**E**).

**Figure 6:**
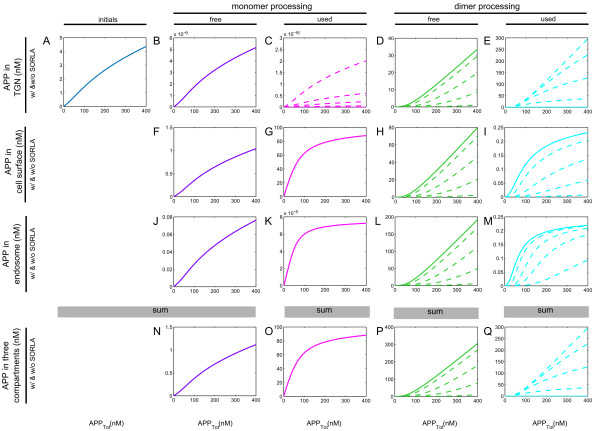
**Distribution of free and used APP within monomer and dimer processing in each compartment.** Simulations of the influence of intermediate levels of SORLA on APP processing into the amounts of APP concentrations in the TGN (**A-E**), at the cell surface (**F-I**), in the endosomes (**J-M**), and in all the three compartments (**N-Q**). The term “used” refers to the complex formation of (i) APP and SORLA in the TGN, (ii) APP and α-secretase at the cell surface, and (iii) APP and β-secretase in the endosomes. Wherein, the term “free” refers to the APP that is not bound in the respective compartments. There are five intermediate levels of SORLA, namely, 0% (solid line), 3%, 12%, 30%, and 100% (dashed line) of SORLA_Tot_ (where SORLA_Tot_ = 2.43 x 10^5^ fmol). If the dashed line is not seen in a plot, it is because the solid and dashed lines are superimposed. In the first column, there shows the amount of initial APP concentrations that are left free from the monomer and dimer processing (**A**). Next, in the second and third columns, the amounts of APP concentrations that are free and used in monomer processing are shown, respectively. Then, in the last two columns, the amounts of APP concentrations that are free and used in dimer processing are also given. Notice that SORLA has minimal or no influence on APP in monomer processing (2nd and 3rd columns); conversely, SORLA shows strong influence on APP in dimer processing (last two columns).

**Figure 7:**
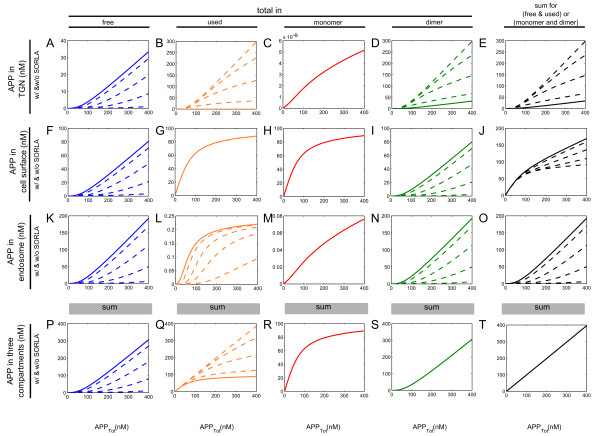
**Total amount of APP that is free, used, in monomer processing, and in dimer processing in each compartment.** Simulations of the influence of intermediate levels of SORLA on APP processing into the amounts of APP concentrations in the TGN (**A-E**), at the cell surface (**F-J**), in the endosomes (**K-O**), and in all the three compartments (**P-T**). There are five intermediate levels of SORLA, namely, 0% (solid line), 3%, 12%, 30%, and 100% (dashed line) of SORLA_Tot_ (where SORLA_Tot_ = 2.43 x 10^5^ fmol). When there is only solid line in a plot, it is because solid and dashed lines are superimposed. Note that the term “used” refers to the complex formation of (i) APP and SORLA in the TGN, (ii) APP and α-secretase at the cell surface, and (iii) APP and β-secretase in the endosomes. Wherein, the term “free” refers to the APP that is not bound in the respective compartments. The first two columns show the total amount of APP concentrations that is free and used. While in the third and fourth columns, the total amount of APP concentrations in monomer and in dimer processing are shown, respectively. Each line in the last column has double meaning: (i) the sum of the corresponding amount of APP concentrations shown in the first two columns, or (ii) the sum of the respective amount of APP concentrations shown in the third and fourth columns. The plots aligned along the first column show that SORLA significantly decreases the total amount of free APP concentrations in each compartment. The plots in the second column show that the total amount of APP concentrations in the TGN increases significantly (**B**), while unaffecting and minimally decreases those at the cell surface (**G**) and in the endosomes (**L**), respectively. As for the plots in the third column, they show that SORLA has no influence on the total amount of APP concentrations in monomer processing. Moreover to the plots in the fourth column, it is observed that as the level of SORLA concentration in dimer processing increases, the total amount of APP concentrations in the TGN also increases (**D**), while those at the cell surface (**I**) and in the endosomes decreases (**N**).

Up to this point, we only showed simulations of our model in the two most extreme scenarios: with no (Figure 2A and B) or high levels of SORLA activity (Figure 2C and D). However, subtle alterations of SORLA concentration are likely to be more relevant for the determination of its influence in APP processing pathways. Accordingly, we adapted our multi-compartment model to intermediate concentrations of SORLA. As shown in Figures 4, 5, 6 and 7 the simulations are all in dependence of three intermediate SORLA expression levels, namely, 3%, 12%, and 30% of SORLA_Tot_.

Remarkably, we observed in Figure 4 that the simulations in dependence of the three intermediate SORLA expression levels are either “spread” (as in Figure 4A and Figure 4D) or “clustered” (as in Figure 4B and Figure 4C) into the two most extreme scenarios of SORLA concentration. This came as a surprised because the dose–response kinetics of total sAPPα production in dependence of the intermediate SORLA expression levels (Figure 4A) is expected to be “clustered” like that of sAPPβ (Figure 4B). Likewise in the case of the amount of APP bound to SORLA in monomer (Figure 4C) and in dimer processing (Figure 4D). We say that the simulations are “clustered” when

$${\text{X}}_{Y}\approx (X_{100\%}-X_{0\%})\cdot Y+X_{0\%}$$

where *Y* = {3%, 12%, 30%}, and *X* denotes the amount of concentration at a given percentage value of SORLA_Tot_ that is specified by its subscript. Otherwise, we say that the simulations are “spread”.

Next, we investigated what leads to the observation made in Figure 4, in dependence of the intermediate SORLA expression levels.

### SORLA indirectly affects the dynamical behavior of the β-secretase but not that of α-secretase

First, we analyzed the simulations of the influence of intermediate levels of SORLA on APP processing on the amount of α-secretase (Figure 5A-F) and β-secretase (Figure 5) concentration. In Figure 5, the term “used” refers to the complex formation of the secretases and APP, while the term “free” refers to the secretases that are not bound in a complex.

The total amount of α-secretase and the total amount of β-secretase were assumed to be constant (depicted by the black lines in Figure 5E-F and Figure 5K-L, respectively). Due to the conservation law assumption, the total amount of each secretase in each subcompartment is conserved (i.e. α_monomer_ and β_monomer_ depicted by red lines in Figure 5F and Figure 5L; α_dimer_ and β_dimer_ depicted by green lines in Figure 5F and Figure 5L). Consequently, the total amount of each secretase in the whole system was thus also conserved (α_Tot_ and β_Tot_ shown by the black lines in Figure 5E-F and Figure 5K-L, respectively).

The simulations of the influence of intermediate levels of SORLA on APP processing on the amount of α-secretase (Figure 5A-F) concentration showed that (i) there are more α-secretases that were used (Figure 5C) than left free (Figure 5A) in monomer processing, (ii) there are more α-secretases that are left free (Figure 5B) than used (Figure 5D) in the dimer processing, (iii) the total amount of α-secretase that is free and used (blue and orange lines in Figure 5E, respectively) is dominated by the corresponding amount of α-secretase concentration in dimer (Figure 5B) and in monomer processing (Figure 5C), (iv) SORLA influences the amount of α-secretase concentration in dimer processing (Figure 5B and 5D), but not those in monomer processing (Figure 5A and 5C), and (v) its simulations in dependence of the three intermediate SORLA expression levels (Figure 5D) is consistent to that of dose–response kinetics of total sAPPα production (Figure 4A).

The significant difference in the free (Figure 5B) and used (Figure 5D) amounts of α-secretase in dimer processing is a consequence of the large amount of α-secretase used in monomer processing (shown in Figure 5C). As the total amount of the APP concentration increases (from 0 nM to 400 nM), the amount of α-secretase, free in dimer processing, (Figure 5B) decreases, while the amount of α-secretase used in monomer processing (Figure 5C) increases. As the amount of SORLA concentration increases, the curves representing the secretases move from solid to dashed lines. SORLA does affect α-secretase in dimer processing (Figure 5B and 5D): those used in dimer processing decreases (Figure 5D), while those that are free in dimer processing increases (Figure 5B). In the later figure, the increase is not obvious because the amount of change is so small as compared to the concentration values of α-secretase.

As for the influence of intermediate levels of SORLA on APP processing on the amount of β-secretase (Figure 5G-L) concentration, the simulations showed that (i) there are more β-secretases that are left free (Figure 5G) than used (Figure 5I) in monomer processing, (ii) SORLA has no influence on β-secretase in monomer processing (Figure 5G and Figure 5I), (iii) SORLA alters the dynamical behaviors of β-secretase in dimer processing (Figure 5H and Figure 5J), (iv) the total amount of β-secretase that is free and used (blue and orange lines in Figure 5K, respectively) is dominated by the amount of β-secretase concentration in dimer processing (Figure 5H and Figure 5J, correspondingly), and (v) its simulations in dependence of the three intermediate SORLA expression levels (Figure 5H and Figure 5J) is consistent to that of dose–response kinetics of total sAPPβ production (Figure 4B). The curves for beta-secretase with SORLA (dashed lines in Figure 5H) are greater in values as compared to those without SORLA (solid line in Figure 5H), as a consequence of SORLA’s influence on beta-secretase that is used in dimer processing (Figure 5J).

When a comparison is made between the total amount of α- and β-secretase concentration that is free (blue lines in Figure 5E and Figure 5K) and used (orange lines in Figure 5E and Figure 5K) in dependence of the three intermediate SORLA expression levels, we observed that the total amount of β-secretase concentration for both free and used deviated (Figure 5K), which was not the case for α-secretase (Figure 5E). This observation suggested that SORLA is indirectly affecting the dynamics of β-secretase but not that of α-secretase. This result supports the hypothesis presented by Schmidt el al. [14]: “the global–local estimation of the parameter values in the model suggested a yet unidentified biological process whereby SORLA might indirectly affect the β-secretase, but not with the α-secretase”. The present result therefore clarifies what was unidentified in our previous study [14].

With SORLA concentration greater than the estimated total amount of SORLA concentration (i.e. SORLA_Tot_ = 2.43 x 10^5^ fmol), we arrived at Figure S1 shown in Additional file 1: Figures S1D and S1J show that for a very large amount of SORLA_Tot_ (greater than 1 x SORLA_Tot_ for α-secretase and greater than 10 x SORLA_Tot_ for β-secretase), the amount of α- and β-secretase are barely “used”. Consequently, the amount of α- (Figure B) and β-secretase (Figure H) are all “free” in dimer processing, and there will be no sAPP products produced in dimer processing.

### SORLA is more influential in dimer processing than in monomer processing

We also investigated the amount of APP concentrations that is either free or used, in monomer or in dimer processing, and which is in the TGN, at the cell surface or in the endosomes (Figure 6). The term “used” refers to the complex formation of (i) APP and SORLA in the TGN, (ii) APP and α-secretase at the cell surface, and (iii) APP and β-secretase in the endosomes. Wherein, the term “free” refers to the APP that is not bound in the respective compartments.

First, we showed the simulations of the amount of APP concentrations that is free or used in monomer and in dimer processing. The simulations under dimer processing showed that the amount of APP concentrations that is free or used in each compartment were significantly affected by the presence of SORLA (last two columns of Figure 6: Figure 6D-E, Figure 6H-I, Figure 6L-M, and Figure 6P-Q), as compared to those under monomer processing (first three columns of Figure 6: Figure 6A-C, Figure 6F-G, Figure 6J-K, and Figure 6N-O). In particular, one observes from the simulations that the amount of APP concentrations that is used to bind with SORLA in dimer processing of the TGN tremendously increases from 0 M to at most 300 nM (Figure 6E), wherein those in monomer processing are so small that they can be neglected (Figure 6C). Consequently, SORLA decreases the amount of APP concentrations that is free or used at the cell surface and in the endosomes (Figure 6H-I and Figure 6L-M, respectively). Also, the total amount of APP concentrations in dimer processing is dominated by the total amount of free APP in the absence of SORLA and by the total amount of used APP in the presence of SORLA (depicted by the two outermost lines in Figure 6P and Figure 6Q).

Next, in each compartment, the simulations for the total amount of APP concentrations that is free, used, in monomer processing, or in dimer processing, are shown in Figure 7. Consistent to our previous observation (Figure 6), the simulations for total amount of APP concentrations in monomer processing for the three different compartments (3^rd^ column of Figure 7) were not influenced by SORLA, while those in dimer processing were affected by the presence of SORLA (4^th^ column of Figure 7). Moreover, the simulations, in the first two columns of Figure 7, also showed that the presence of SORLA in the TGN decreases the total amount of free APP (Figure 7P), and increases the total amount of used APP (Figure 7Q). In particular to the total amount of used APP under the influence of SORLA, it is (i) enormously increased in the TGN (Figure 7B), (ii) not affected at the cell surface (Figure 7G), and (iii) reduced by at most half in the endosomes (Figure 7L). Taken together, the presence of SORLA increases the total amount of APP concentrations in the TGN (Figure 7E), and subsequently decreases the total amount of APP concentrations at the cell surface (Figure 7J) and in the endosomes (Figure 7O).

The simulations for the total amount of APP concentrations in monomer processing (Figure 7R), in dimer processing (Figure 7S), and in both monomer and dimer processing (Figure 7T) show that a conservation law was assumed for APP in monomer and in dimer processing. Above all, one observes that there are more APP concentrations in dimer processing (Figure 7S) than in monomer processing (Figure 7R).

### The spread and clustering of SORLA expression levels

As noted in the subsection, *Intermediate levels of SORLA*, the simulations show that SORLA expression levels are either “spread” (Figure 4A) or “clustered” (Figure 4B). This is most likely due to the effect of SORLA on the processing of APP dimer. With respect to the total amount of APP, the amount of APP concentrations (Figure 6I) and α-secretase concentrations (Figure 5D) that are “used” at the cell surface in dimer processing “spread”. Considering the relevance of APP and α-secretase at the cell surface to the production of sAPPα, the observations thus suggest the “spread” observed in Figure 4A for sAPPα. Similarly for the “clustering” observed in Figure 4B for sAPPβ, it is a consequence of the “clustering” that is observed on APP (Figure 6M) and β-secretase (Figure 5J) that are “used” in the endosome in dimer processing, which are relevant in producing sAPPβ. Moreover, the change from “spread” at the cell surface (Figure 4A) to “clustered” in the endosome (Figure 4B) is probably due to the indirect influence of SORLA on the dynamical behavior of β-secretase that is observed in Figure 5.

### Effects of different SORLA concentrations in switching sAPPα and sAPPβ from preferred dimer-to-monomer processing

**Figure 8:**
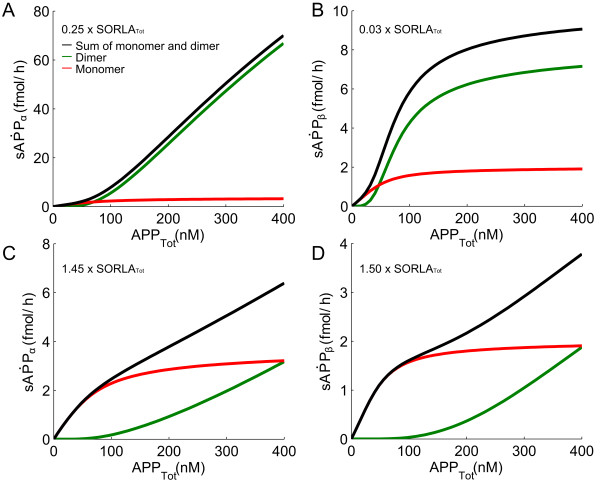
**Switch from preferred dimer-to-monomer processing.** Simulations of the influence of SORLA on APP processing into sAPPα (**A, C**) and sAPPβ (**B, D**) are shown (where SORLA_Tot_ = 2.43 x 10^5^ fmol). Total amount of products produced from both processing (black line) as well as the products produced from dimer (green line) and monomer (red line) processing are indicated for each simulation. A switch from preferred dimer-to-monomer processing is seen at 25% of SORLA_Tot_ for α-secretase (**A**) and at 3% of SORLA_Tot_ for β-secretase (**B**). The amount of product obtained from ‘red’ monomer is greater than that of ‘green’ dimer is observed at 145% of SORLA_Tot_ for α-secretase (**C**) and at 150% of SORLA_Tot_ for β-secretase (**D**).

Lastly, in Figure 8, are given simulations of the influence of SORLA on APP processing into sAPPα (Figure 8A and Figure 8C) and sAPPβ (Figure 8B and Figure 8D). The simulations show that the switch from preferred dimer-to-monomer processing is observed at 25% of SORLA_Tot_ for α-secretase (Figure 8A) and at 3% of SORLA_Tot_ for β-secretase (Figure 8B), where SORLA_Tot_ equals 2.43 x 10^5^ fmol. In agreement with the study performed by Schmidt and colleagues [14] previously, we therefore find that the switch from cooperative (dimer) to less efficient non-cooperative (monomer) processing occurs at small amount of SORLA concentration. Moreover, the end product obtained from monomer processing dominates the total amount of end product at 145% of SORLA_Tot_ for α-secretase (Figure 8C) and at 150% of SORLA_Tot_ for β-secretase (Figure 8D). In connection to what we observed in Figure 4 for the simulations of the influence of intermediate levels of SORLA on APP processing into sAPPα (Figure 4A) and sAPPβ (Figure 4B), these two sets of results (Figure 4 and Figure 8) suggest that SORLA reduces the products produced in non-amyloidogenic and amyloidogenic pathways of APP processing at different rate.

## Conclusions

Our multi-compartment model is an extension of the single-compartment model that was established by Schmidt and colleagues [14] previously. To our knowledge, this is the first multi-compartmental model developed to analyze APP processing in the context of Alzheimer’s disease. In addition, our model represents the regulated trafficking of APP by SORLA through the intracellular compartments, which critically affects amyloidogenic and non-amyloidogenic processing pathways [20]. Our model was established to answer questions that arose from a study based on a single-compartment model [14].

The first question that emerged concerned the relative contributions of SORLA to monomer and in dimer processing. In the study conducted by Schmidt and colleagues [14], we showed that SORLA influenced the combined products obtained by monomer and dimer processing. However, limited by the structure of the single-compartment model, we were not able to investigate the relative contribution of SORLA in monomer and in dimer processing. Herein, using our multi-compartment model, we showed that the decrease in total amounts of sAPPα and sAPPβ is mainly due to the influence of SORLA in dimer processing. This observation confirms previous hypothesis that SORLA, prevent oligomerization of APP, eliminating the preferred substrates for secretases.

The second question was how does SORLA affect the dynamics of β-secretase? In the study conducted by Schmidt and colleagues [14], it was suggested that there is an indirect effect of the SORLA receptor on the β-secretase, which contributes to the regulation of amyloidogenic processing in the context of an intact cell. However, in order for the single-compartment model to closely resemble the experimental data, the model required a local parameter estimate for β-secretase activity in the presence or absence of SORLA. Through our multi-compartment model, where all parameters are estimated globally, we now confirmed that SORLA affects the interaction between APP and β-secretase, but not that of APP with α-secretase. A previous study suggested that SORLA directly interacts with β-secretase, preventing access of the enzyme to its substrate APP [15]. While our simulations confirm an important influence of SORLA on β-secretase, this influence may also be indirect, for example by effecting trafficking of cofactors essential for enzyme activity. An indirect effect of SORLA is in line with findings that the receptor does not impair β-secretase activity in cell-free assays [14].

In addition, we investigated the regulated trafficking of APP by SORLA in monomer and dimer processing, considering several cellular compartments, including TGN, cell surface, and endosomes. Simulations of our multi-compartment model showed that SORLA increases the total amount of APP concentrations in the TGN (Figure 7E) and subsequently decreases the total amount of APP at the cell surface (Figure 7J) and endosomes (Figure 7O). In agreement with Andersen and Willnow [21], this result suggests that an over-expression of SORLA prevents the localization of APP from the TGN to the cell surface and to the endosomes, whereby an over-expression of SORLA decreases the products produced in the amyloidogenic and non-amyloidogenic pathways of APP processing. Furthermore, our study confirmed that SORLA is more influential in dimer than in monomer processing. This observation is in line with our previous model that APP dimers represent the preferred substrate for α- and β-secretase as they enable cooperativity in substrate binding [14]. Taken together, data obtained both in single and in multi-compartment models strongly suggested that depletion of APP dimer processing represents a major molecular mechanism in the pathology of Alzheimer’s disease.

Our multi-compartment model was used to simulate pathological situations involving APP under different level of SORLA concentration. Our model can also be used as a kinetic-dynamic model to study the effects of SORLA on α- and β- secretase. Moreover, we observed that as the amount of SORLA concentration increases, there is a relatively large decrease in the production rate of sAPPα as compared to that of sAPPβ (Figure 4 and Figure 8).

Using this refined model together with the chosen set of estimated parameter values (shown in Additional file 1: Table S4), our results suggest the following biological implications of SORLA: (1) Decrease in total amounts of sAPP products is mainly due to the large amount of SORLA concentration in dimer processing (2.43 x 10^5^ fmol), and not to the small amount of SORLA concentration in monomer processing (1.23 x 10^1^ fmol). (2) SORLA indirectly affects the dynamical behavior of the β-secretase but not that of α-secretase. The receptor targets β-secretase, the enzyme responsible for initial amyloidogenic cleavage. This finding represents a major conceptual advance in our understanding of the complex processes in APP processing and supports initial biochemical data that SORLA can bind to β-secretase [15]. (3) SORLA is more influential in dimer processing that in monomer processing, which confirmed our initial hypothesis that blockade of APP dimerization is an important aspect of SORLA action on AD.

In future studies, we will extend this model by including additional cleavage activity by γ-secretase in monomer and in dimer processing. Cleavage of APP by γ-secretase leads to the formation of Aβ peptides, which is the main pathology of Alzheimer’s disease. The ultimate goal is therefore to establish a model that will test the potential effects of SORLA on APP processing in the context of AD therapy.

## Methods

### Model equations

Based on the biochemical network (Figure 1), we established ODEs that describe temporal changes of molecular numbers for the network components as a function of interaction and cleavage processes, such that the changes with large numbers of molecules can be assumed to be smooth. The complete formulation of the model, realized as set of ODEs, can be found in the Additional file 1. Also, the notations used in the equations are described in Additional file 1: Table S2.

Recall that the components in our multi-compartment model, separated by the three different compartments and two different subcompartments, are annotated differently. Distinctly labeling them allows differentiation and comparison of the results generated from these multi-compartment. Consequently, we could distinguish the level of influence of SORLA between the monomeric and the dimeric form of APP processing, and also determine in which form of APP processing is SORLA more influential.

Herein, we showed a series of assumptions that allowed the reduction of the equations.

Firstly, we started with the transportation of APP, α-, and β-secretase among the three compartments and between the two subcompartments. Recall that the (i) APP-monomers and APP-dimers are transported from the TGN to the cell surface, and are then further transported to the endosomes, (ii) monomeric forms of APP, SORLA, α-secretase, and β-secretase, within the two subcompartments, are annotated differently, and (iii) components within the two subcompartments are linked to each other via *APP*_*init*_, *α*_*init*_, and *β*_*init*_. These properties are reflected by introducing the following biochemical reactions: For APP,

$$\begin{array}{c} APP_{\mathrm{init}}\overset{K_{G1}}{\to }APP_{G1}\\ APP_{\mathrm{init}}\overset{K_{G2}}{\to }APP_{G2}\\ APP_{G1}\overset{K_{CS1}}{\to }APP_{CS1}\\ APP_{G2d}\overset{K_{CS2}}{\to }APP_{CS2d} \end{array}$$

and for the secretases,

$$\begin{array}{c} \alpha_{\mathrm{init}}\overset{K_{C1}}{\to }\alpha_{1}\\ \alpha_{\mathrm{init}}\overset{K_{C2}}{\to }\alpha_{2}\\ \beta_{\mathrm{init}}\overset{K_{B1}}{\to }\beta_{1}\\ \beta_{\mathrm{init}}\overset{K_{B2}}{\to }\beta_{2} \end{array}$$

where the second reactant is assumed to be in quasi-equilibrium with the first reactant. Without loss of generality, the concentration of the second reactant is related to the first reactant by an ordinary equilibrium expression, such as

(1) $$\left(\begin{array}{c} APP_{G1}=K_{G1}\cdot APP_{\mathrm{init}}\\ APP_{G2}=K_{G2}\cdot APP_{\mathrm{init}}\\ APP_{CS1}=K_{CS1}\cdot APP_{G1}\\ APP_{CS2d}=K_{CS2}\cdot APP_{G2d}\\ \alpha_{1}=K_{C1}\cdot \alpha_{\mathrm{init}}\\ \alpha_{2}=K_{C2}\cdot \alpha_{\mathrm{init}}\\ \beta_{1}=K_{B1}\cdot \beta_{\mathrm{init}}\\ \beta_{2}=K_{B2}\cdot \beta_{\mathrm{init}} \end{array}\right\}$$

where $K_{G1}=k_{g1}/k_{-g1}$, $K_{G2}=k_{g2}/k_{-g2}$, $K_{CS1}=k_{cs1}/k_{-cs1}$, $K_{CS2}=k_{cs2}/k_{-cs2}$, $K_{C1}=k_{c1}/k_{-c1}$, $K_{C2}=k_{c2}/k_{-c2}$, $K_{B1}=k_{b1}/k_{-b1}$, and $K_{B2}=k_{b2}/k_{-b2}$.

As for

$$\begin{array}{c} APP_{CS1}\begin{array}{c} \overset{k_{e1}}{\to }\\ \underset{k_{-e1}}{\leftarrow } \end{array}APP_{E1}\\ APP_{CS2d}\begin{array}{c} \overset{k_{e2}}{\to }\\ \underset{k_{-e2}}{\leftarrow } \end{array}APP_{E2d} \end{array}$$

the ratio of the association constant is taken into consideration. This assumption permits the two reactants to be related to each other by an association constant, such that

(2) $$\left(\begin{array}{c} APP_{E1}=K_{E1}\cdot APP_{CS1}\\ APP_{E2d}=K_{E2}\cdot APP_{CS2d} \end{array}\right\}$$

where $K_{E1}=k_{e1}/k_{-e1}$ and $K_{E2}=k_{e2}/k_{-e2}$.

Secondly, the monomeric forms of APP, α-, and β-secretase in dimer processing undergo dimerization. In other words, two monomeric forms of APP, α-, or β-secretase are dimerized, and a dimeric form of APP, α-, or β-secretase is dissociated, as shown by the reactions below:

$$\begin{array}{cc} APP_{G2}+APP_{G2} & \begin{array}{c} \overset{k_{g3}}{\to }\\ \underset{k_{-g3}}{\leftarrow } \end{array}APP_{G2d}\\ \alpha_{2}+\alpha_{2} & \begin{array}{c} \overset{k_{c3}}{\to }\\ \underset{k_{-c3}}{\leftarrow } \end{array}\alpha_{2d}\\ \beta_{2}+\beta_{2} & \begin{array}{c} \overset{k_{b3}}{\to }\\ \underset{k_{-b3}}{\leftarrow } \end{array}\beta_{2d} \end{array}$$

As we took into account the association constant of each reaction above, it allows us to have the following representation of the equation:

(3) $$\left(\begin{array}{c} APP_{G2d}=K_{G3}\cdot APP_{G2}^{2}\\ \alpha_{2d}=K_{C3}\cdot \alpha_{2}^{2}\\ \beta_{2d}=K_{B3}\cdot \beta_{2}^{2} \end{array}\right\}$$

where $K_{G3}=k_{g3}/k_{-g3}$, $K_{C3}=k_{c3}/k_{-c3}$, and $K_{B3}=k_{b3}/k_{-b3}$.

Up to this point, we had established equations that describe the relationship of APP, α-, or β-secretase, which are defined in the three different compartments.

Thirdly, at the cell surface, APP interacts with α-secretase through the following reactions

$$\begin{array}{c} APP_{CS1}+\alpha_{1}\begin{array}{c} \overset{k_{5}}{\to }\\ \underset{k_{-5}}{\leftarrow } \end{array}C_{APPCS1\alpha 1}\overset{k_{6}}{\to }sAPP\alpha_{1}+C83_{1}+\alpha_{1}\\ APP_{CS2d}+\alpha_{2d}\begin{array}{c} \overset{k_{51}}{\to }\\ \underset{k_{-51}}{\leftarrow } \end{array}C_{APPCS2d\alpha 2d}\overset{k_{61}}{\to }2\cdot sAPP\alpha_{2}+C83_{2d}+\alpha_{2d} \end{array}$$

Whereas, in the endosomes, the following reactions take place:

$$\begin{array}{c} APP_{E1}+\beta_{1}\begin{array}{c} \overset{k_{3}}{\to }\\ \underset{k_{-3}}{\leftarrow } \end{array}C_{APPE1\beta 1}\overset{k_{4}}{\to }sAPP\beta_{1}+C99_{1}+\beta_{1}\\ APP_{E2d}+\beta_{2d}\begin{array}{c} \overset{k_{31}}{\to }\\ \underset{k_{-31}}{\leftarrow } \end{array}C_{APPE2d\beta 2d}\overset{k_{41}}{\to }2\cdot sAPP\beta_{2}+C99_{2d}+\beta_{2d} \end{array}$$

For the ODEs of the complexes above (found in Additional file 1), a quasi-steady state can be assumed. This allows the complexes to be represented as:

(4) $$\left(\begin{array}{c} C_{APPCS1\alpha 1}=\alpha_{1}\cdot APP_{CS1}/K_{M\alpha 1}\\ C_{APPCS2d\alpha 2d}=\alpha_{2d}\cdot APP_{CS2d}/K_{M\alpha 2d}\\ C_{APPE1\beta 1}=\beta_{1}\cdot APP_{E1}/K_{M\beta 1}\\ C_{APPE2d\beta 2d}=\beta_{2d}\cdot APP_{E2d}/K_{M\beta 2d} \end{array}\right\}$$

where $K_{M\alpha 1}=(k_{-5}+k_{6})/k_{5}$, $K_{M\alpha 2d}=(k_{-51}+k_{61})/k_{51}$, $K_{M\beta 1}=(k_{-3}+k_{4})/k_{3}$, and $K_{M\beta 2d}=(k_{-31}+k_{41})/k_{31}$. These equations for the complexes are turned into the ODEs that describe the formation of the sAPP products: the ODEs of the sAPP products in the monomeric form of APP processing can be rewritten as

(5) $$\left(\begin{array}{c} \overset{\cdot }{sAPP\alpha_{1}}=\frac{k_{6}\cdot \alpha_{1}\cdot APP_{CS1}}{K_{M\alpha 1}}\\ \overset{\cdot }{sAPP\beta_{1}}=\frac{k_{4}\cdot \beta_{1}\cdot APP_{E1}}{K_{M\beta 1}} \end{array}\right\}$$

whereas those in the dimeric form of APP processing can be written in the following form

(6) $$\left(\begin{array}{c} \overset{\cdot }{sAPP\alpha_{2}}=\frac{k_{61}\cdot \alpha_{2d}\cdot APP_{CS2d}}{K_{M\alpha 2d}}\\ \overset{\cdot }{sAPP\beta_{2}}=\frac{k_{41}\cdot \beta_{2d}\cdot APP_{E2d}}{K_{M\beta 2d}} \end{array}\right\}$$

From Equations (5) and (6), we obtain

(7) $$\left(\begin{array}{c} \overset{\cdot }{sAPP\alpha_{\mathrm{Tot}}}=\overset{\cdot }{sAPP\alpha_{1}}+\overset{\cdot }{sAPP\alpha_{2}}\\ \overset{\cdot }{sAPP\beta_{\mathrm{Tot}}}=\overset{\cdot }{sAPP\beta_{1}}+\overset{\cdot }{sAPP\beta_{2}} \end{array}\right\}$$

Fourthly, recall that *APP*_*G1*_ binds to *SORLA*_*G1*_ with a binding affinity of *K*_*S1*_ in monomer processing, whereas *APP*_*G2*_ to *SORLA*_*G2*_ with a different binding affinity *K*_*S2*_ in dimer processing. Note that $K_{S1}=k_{s1}/k_{-s1}$ and $K_{S2}=k_{s2}/k_{-s2}$. These properties are reflected in the biochemical reactions below:

$$\begin{array}{l} APP_{G1}+SORLA_{G1}\begin{array}{c} \overset{k_{s1}}{\to }\\ \underset{k_{-s1}}{\leftarrow } \end{array}C_{APPG1SORLAG1}\\ APP_{G2}+SORLA_{G2}\begin{array}{c} \overset{k_{s2}}{\to }\\ \underset{k_{-s2}}{\leftarrow } \end{array}C_{APPG2SORLAG2} \end{array}$$

We then took into consideration the rapid-equilibrium assumption for the *C*_*APPG1SORLAG1*_ and *C*_*APPG2SORLAG2*_ complexes, which gives us

(8) $$\left(\begin{array}{c} C_{APPG1SORLAG1}=K_{S1}\cdot APP_{G1}\cdot SORLA_{G1}\\ C_{APPG2SORLAG2}=K_{S2}\cdot APP_{G2}\cdot SORLA_{G2} \end{array}\;\right\}$$

Lastly, the ODEs include conservation laws for the molecule numbers of enzymes and substrates. Herein, we took into account Equations (1) to (4) and (8) that are shown previously. For α-secretase and β-secretase, conservation law assumption leads to

(9) $$\left(\begin{array}{l} \alpha_{\mathrm{Tot}}=\alpha_{\mathrm{init}}+\alpha_{\mathrm{monomer}}+\alpha_{\text{dimer}}\\ \beta_{\mathrm{Tot}}=\beta_{\mathrm{init}}+\beta_{\mathrm{monomer}}+\beta_{\text{dimer}} \end{array}\right\}$$

where

(10) $$\left(\begin{array}{c} \alpha_{\mathrm{monomer}}=\alpha_{1}+C_{APPCS1\alpha 1}\\ \alpha_{\text{dimer}}=\alpha_{2}+\alpha_{2d}+C_{APPCS2d\alpha 2d}\\ \beta_{\mathrm{monomer}}=\beta_{1}+C_{APPE1\beta 1}\\ \beta_{\text{dimer}}=\beta_{2}+\beta_{2d}+C_{APPE2d\beta 2d} \end{array}\right\}$$

For SORLA,

(11) $${\text{SORLA}}_{\text{Tot}}={\text{SORLA}}_{\text{monomer}}+{\text{SORLA}}_{\text{dimer}}$$

where

(12) $$\left(\begin{array}{c} SORLA_{\mathrm{monomer}}=SORLA_{G1}+C_{APPG1SORLAG1}\\ SORLA_{\dim er}=SORLA_{G2}+C_{APPG2SORLAG2} \end{array}\;\right\}$$

Moreover, the ODEs also include conservation of the APP substrate. This leads to the following representation:

(13) $$APP_{\mathrm{Tot}}=APP_{\mathrm{init}}+APP_{\mathrm{monomer}}+APP_{\text{dimer}}$$

where

(14) $$\left(\begin{array}{c} APP_{\mathrm{monomer}}(APP_{\mathrm{init}},\alpha_{\mathrm{init}},\beta_{\mathrm{init}})=APP_{G1}+APP_{CS1}+APP_{E1}\\ +C_{APPCS1\alpha 1}+C_{APPE1\beta 1}\\ APP_{\text{dimer}}(APP_{\text{init}},\alpha_{\mathrm{init}},\beta_{\text{init}})=APP_{G2}+APP_{G2d}+APP_{CS2d}+APP_{E2d}\\ +C_{APPCS2d\alpha 2d}+C_{APPE2d\beta 2d} \end{array}\;\right\}$$

in the absence of SORLA, whereas

(15) $$\left(\begin{array}{c} APP_{\mathrm{monomer}}(APP_{G1},\alpha_{1},\beta_{1})=APP_{G1}+APP_{CS1}+APP_{E1}+\\ C_{APPCS1\alpha 1}+C_{APPE1\beta 1}+C_{APPG1SORLAG1}\\ APP_{\text{dimer}}(APP_{G2},\alpha_{2},\beta_{2})=APP_{G2}+APP_{G2d}+APP_{CS2d}+APP_{E2d}+\\ C_{APPCS2d\alpha 2d}+C_{APPE2d\beta 2d}+C_{APPG2SORLAG2} \end{array}\;\right\}$$

in the presence of SORLA.

Below, we discuss in more detail the properties behind equations (9)-(15): (I) each conserved equation is a function of free reactant and of reactant bound in the complexes. Note that the reactant can be the APP, SORLA, α-, or β-secretase. (II) Each *Reactant*_*Tot*_ function is represented differently in the presence and in the absence of SORLA. In particular, each *Reactant*_*Tot*_ function without SORLA is transcribed as function of *APP*_*init*_, *α*_*init*_*,* and *β*_*init*_, whereas those with SORLA are transcribed as function of *APP*_*G1*_*, APP*_*G2*_*, α*_*1*_*, α*_*2*_*, β*_*1*_*,* and *β*_*2*_. (III) The amount of the *Reactant*_*init*_ in each *Reactant*_*Tot*_ with SORLA is set to be the same as that calculated from the corresponding *Reactant*_*Tot*_ without SORLA. (IV) Similarly, the total amount of *Reactant*_*monomer*_ and *Reactant*_*dimer*_ in each *Reactant*_*Tot*_ with SORLA are equivalent to those calculated from the corresponding *Reactant*_*Tot*_ without SORLA. (V) The amount of *SORLA*_*Tot*_, *α*_*Tot*_, and *β*_*Tot*_ are assumed to be constant for different amount of *APP*_*Tot*_ concentration. This assumption is based on the experimental design applied on the series of dose–response data [14] that are used in this study. (VI) Without loss of generality, *SORLA*_*monomer*_ and *SORLA*_*dimer*_ are also assumed to be constant for different amount of *APP*_*Tot*_ concentration.

The properties defined above are, in particular, necessary and important. Without those properties, the presence of SORLA in monomer processing will not only affect the monomeric form of APP processing, it will also indirectly influence the dimeric form of APP processing, and vice versa. As such, it defeated the main purpose of this study, which is to differentiate the level of influence of SORLA in monomer and in dimer processing.

### Model parameter estimation

The development of the model described in the previous section reduced the number of free parameters from 77 to 27. The reduced number of parameter values of the model were estimated by nonlinear optimization such that the model simulations fit four biological independent dose–response series without SORLA (a total of *N* = 64 experimental data points) and five biological independent dose–response series with SORLA (also a total of *N* = 64 experimental data points). We looked for a set of parameter values that minimizes the weighted least squares function of *APP*_*Tot*_ with SORLA, and *sAṖPα*_*Tot*_ and *sAṖPβ*_*Tot*_ in regardless of SORLA (Equation (7)). On account of the different orders of magnitude of the experimental values of APP, sAPPα, and sAPPβ, weights were assigned such that the influence of each data set in the process of optimization will be equal. The weights are defined as

(16) $$\begin{array}{c} w_{a}=\frac{\sum_{k=1}^{N}\dot{sAPP\alpha_{k}^{E}}}{N},\\ w_{b}=\frac{\sum_{k=1}^{N}\dot{sAPP\beta_{k}^{E}}}{N},\\ w_{\mathrm{aS}}=\frac{\sum_{k=1}^{N}\dot{sAPP\alpha_{S,k}^{E}}}{N},\\ w_{\mathrm{bS}}=\frac{\sum_{k=1}^{N}\dot{sAPP\beta_{S,k}^{E}}}{N},\\ w_{\mathrm{appS}}=\frac{\sum_{k=1}^{N}APP_{S,k}^{E}}{N} \end{array}$$

where the superscript ‘*E*’ and the subscript ‘*S*’ denotes experimental data points and the influence of SORLA, respectively. The goodness of fit was quantified by calculating the residual value, i.e. the sum of the squared differences between the data and model, divided by a respective weight:

(17) $$residual=\min\sum_{k=1}^{N}\left(\begin{array}{l} \frac{{\left(APP_{S,k}^{E}-APP_{Tot,S,k}\right)}^{2}}{w_{\mathrm{appS}}}+\frac{{\left(\dot{sAPP\alpha_{k}^{E}}-\dot{sAPP\alpha_{Tot,k}}\right)}^{2}}{w_{a}}+\frac{{\left(\dot{sAPP\beta_{k}^{E}}-\dot{sAPP\beta_{Tot,k}}\right)}^{2}}{w_{b}}\\ +\frac{{\left(\dot{sAPP\alpha_{S,k}^{E}}-\dot{sAPP\alpha_{Tot,S,k}}\right)}^{2}}{w_{\mathrm{aS}}}+\frac{{\left(\dot{sAPP\beta_{S,k}^{E}}-\dot{sAPP\beta_{Tot,S,k}}\right)}^{2}}{w_{\mathrm{bS}}} \end{array}\;\right)\text{.}$$

We used the lsqnonlin and fzero functions in the MATLAB optimization toolbox [22] to estimate unknown parameter values. The estimation of parameter values was performed by the steps elaborated in Additional file 1: Table S3.

We performed 500 global estimates, satisfying the condition that all parameter values are positive. Note that none of the parameter values are taken from the literature due to the differences in the experimental methods applied. Most kinetic data available in the literature on α/β-secretase activity were obtained in cell free assays with purified enzyme and artificial peptide substrate. This is in contrast to our model that relies in quantitative data obtained on APP processing in intact cell. Furthermore, the parameter values estimated for our multi-compartment model are expected to differ from that of the single-compartment model by Schmidt and colleagues [14]. Out of the 500 simulation runs, we took the set of estimated parameter values that has the smallest residual value, as shown in Additional file 1: Table S4.

### Experimental materials and methodology

Details about the protocol and assay procedures of the experimental data used in this study can be found in the paper by Schmidt and colleagues [14].

## Competing interests

The authors declare that they have no conflict of interest.

## Authors' contributions

AL initiated the ideas and concepts of the study, carried out the calculations, and performed the simulations. VS and YS participated in the design of the study, formulation of the model, analysis of the results, and editing the manuscript. TW and OW supervised the work and contributed to the writing of the final manuscript. All authors read and approved the final manuscript.

## Supplementary Material

Additional file 1 **Mathematical Modeling.** The complete formulation of the model, realized as set of ODEs. **Table S1 – Variables in the biochemical network.** The table contains the description of the variables that are used in the different compartments of the biochemical network (shown in Figure 1). **Table S2 – Variables and parameters in the mathematical model**. The table contains the unit and the description of the variables and parameters used in the mathematical model. **Table S3 – Simulation steps.** The steps performed for the estimation of parameter values are elaborated here. **Table S4 - Estimated parameter values for Figure**2. The table shows a set of estimated parameter values, which has the lowest residual value out of 500 simulation runs. **Figure S1 – Concentration values of the secretases with higher SORLA**_**Tot**_**values.** The figure shows simulations of the influence of intermediate levels of SORLA that are greater that the value of SORLA_Tot_, on the amount of α-secretase and β-secretase concentrations on APP processing. Click here for file
