# Supplementary material for: Multi-compartmental modeling of SORLA’s influence on amyloidogenic processing in Alzheimer’s disease
Source: BMC Syst Biol. 2012 Jun 22;6:74. doi: 10.1186/1752-0509-6-74 (PMC3483162; doi:10.1186/1752-0509-6-74)
Supplement: Additional file 1 — Mathematical Modeling. The complete formulation of the model, realized as set of ODEs. Table S1 – Variables in the biochemical network. The table contains the description of the variables that are used in the different compartments of the biochemical network (shown in Figure 1). Table S2 – Variables and parameters in the mathematical model. The table contains the unit and the description of the variables and parameters used in the mathematical model. Table S3 – Simulation steps. The steps performed for the estimation of parameter values are elaborated here. Table S4 - Estimated parameter values for Figure2. The table shows a set of estimated parameter values, which has the lowest residual value out of 500 simulation runs. Figure S1 – Concentration values of the secretases with higher SORLATotvalues. The figure shows simulations of the influence of intermediate levels of SORLA that are greater that the value of SORLATot, on the amount of α-secretase and β-secretase concentrations on APP processing. [file 1752-0509-6-74-S1.pdf]

ADDITIONAL FILE

Multi-compartmental modeling of SORLA's influence on amyloidogenic processing in Alzheimers disease

Angelyn Lao,* Vanessa Schmidt,† Yvonne Schmitz,* Thomas Willnow,† and Olaf Wolkenhauer*‡

Contents

1 Mathematical Modeling 2
1.1 Kinetic reactions . . . 2
1.2 Ordinary differential equations (ODEs) . . . 2
1.3 Assumptions . . . 4
1.3.1 Quasi-steady state . . . 4
1.3.2 Rapid-Equilibrium . . . 4
1.3.3 Quasi-equilibrium . . . 4
1.3.4 Ratio of association constant . . . 5
1.3.5 Law of conservation . . . 6
1.4 End products . . . 10

2 List of Additional Tables 11
Table S1 . . . 11
Table S2 . . . 12
Table S3 . . . 14
Table S4 . . . 15

3 List of Additional Figures 16
Figure S1 . . . 16

*Department of Systems Biology and Bioinformatics, Institute of Computer Science, University of Rostock,18051 Rostock, Germany
†Max-Delbrück-Center for Molecular Medicine, 13125 Berlin
‡Stellenbosch Institute for Advanced Study (STIAS), Stellenbosch 7600, South Africa

1 Mathematical Modeling

1.1 Kinetic reactions

TGN

$$
\begin{array}{lllcl}
 & & & \xrightarrow{k_{g0}} & APP_{init} \\
APP_{init} & & & \xrightarrow{K_{G1}} & APP_{G1} \\
APP_{init} & & & \xrightarrow{K_{G2}} & APP_{G2} \\
APP_{G2} & + & APP_{G2} & \underset{k_{-g3}}{\overset{k_{g3}}{\rightleftharpoons}} & APP_{G2d} \\
APP_{G1} & + & SORLA_{G1} & \underset{k_{-s1}}{\overset{k_{s1}}{\rightleftharpoons}} & C_{APPG1SORLAG1} \\
APP_{G2} & + & SORLA_{G2} & \underset{k_{-s2}}{\overset{k_{s2}}{\rightleftharpoons}} & C_{APPG2SORLAG2}
\end{array}
$$

cell surface

$$
\begin{array}{lllclcl}
APP_{G1} & & & \xrightarrow{K_{CS1}} & APP_{CS1} & & \\
APP_{G2d} & & & \xrightarrow{K_{CS2}} & APP_{CS2d} & & \\
\alpha_{init} & & & \xrightarrow{K_{C1}} & \alpha_1 & & \\
\alpha_{init} & & & \xrightarrow{K_{C2}} & \alpha_2 & & \\
\alpha_2 & + & \alpha_2 & \underset{k_{-c3}}{\overset{k_{c3}}{\rightleftharpoons}} & \alpha_{2d} & & \\
APP_{CS1} & + & \alpha_1 & \underset{k_{-5}}{\overset{k_5}{\rightleftharpoons}} & C_{APPCS1\alpha1} & \xrightarrow{k_6} & \text{sAPP}\alpha_1 + C83_1 + \alpha_1 \\
APP_{CS2d} & + & \alpha_{2d} & \underset{k_{-51}}{\overset{k_{51}}{\rightleftharpoons}} & C_{APPCS2d\alpha2d} & \xrightarrow{k_{61}} & 2 \cdot \text{sAPP}\alpha_2 + C83_{2d} + \alpha_{2d}
\end{array}
$$

endosome

$$
\begin{array}{lllclcl}
APP_{CS1} & & & \underset{k_{-e1}}{\overset{k_{e1}}{\rightleftharpoons}} & APP_{E1} & & \\
APP_{CS2d} & & & \underset{k_{-e2}}{\overset{k_{e2}}{\rightleftharpoons}} & APP_{E2d} & & \\
\beta_{init} & & & \xrightarrow{K_{B1}} & \beta_1 & & \\
\beta_{init} & & & \xrightarrow{K_{B2}} & \beta_2 & & \\
\beta_2 & + & \beta_2 & \underset{k_{-b3}}{\overset{k_{b3}}{\rightleftharpoons}} & \beta_{2d} & & \\
APP_{E1} & + & \beta_1 & \underset{k_{-3}}{\overset{k_3}{\rightleftharpoons}} & C_{APPE1\beta1} & \xrightarrow{k_4} & \text{sAPP}\beta_1 + C99_1 + \beta_1 \\
APP_{E2d} & + & \beta_{2d} & \underset{k_{-31}}{\overset{k_{31}}{\rightleftharpoons}} & C_{APPE2d\beta2d} & \xrightarrow{k_{41}} & 2 \cdot \text{sAPP}\beta_2 + C99_{2d} + \beta_{2d}
\end{array}
$$

1.2 Ordinary differential equations (ODEs)

APP

$$
\begin{aligned}
\dot{APP}_{init} &= k_{g0} - (k_{g1} + k_{g2}) \cdot APP_{init} + k_{-g1} \cdot APP_{G1} + k_{-g2} \cdot APP_{G2} \\
\dot{APP}_{G1} &= k_{g1} \cdot APP_{init} - (k_{-g1} + k_{cs1}) \cdot APP_{G1} + k_{-cs1} \cdot APP_{CS1} - \\
&\quad k_{s1} \cdot APP_{G1} \cdot SORLA_{G1} + k_{-s1} \cdot C_{APPG1SORLAG1} \\
\dot{APP}_{G2} &= k_{g2} \cdot APP_{init} - k_{-g2} \cdot APP_{G2} + 2 \cdot (k_{-g3} \cdot APP_{G2d} - k_{g3} \cdot APP_{G2}^2) - \\
&\quad k_{s2} \cdot APP_{G2} \cdot SORLA_{G2} + k_{-s2} \cdot C_{APPG2SORLAG2} \\
\dot{APP}_{G2d} &= 2 \cdot (k_{g3} \cdot APP_{G2}^2 - k_{-g3} \cdot APP_{G2d}) - k_{cs2} \cdot APP_{G2d} + k_{-cs2} \cdot APP_{CS2d} \\
\dot{APP}_{CS1} &= k_{cs1} \cdot APP_{G1} - (k_{-cs1} + k_{e1}) \cdot APP_{CS1} + k_{-e1} \cdot APP_{E1} - \\
&\quad k_5 \cdot APP_{CS1} \cdot \alpha_1 + k_{-5} \cdot C_{APPCS1\alpha1} \\
\dot{APP}_{CS2d} &= k_{cs2} \cdot APP_{G2d} - (k_{-cs2} + k_{e2}) \cdot APP_{CS2d} + k_{-e2} \cdot APP_{E2d} - \\
&\quad k_{51} \cdot APP_{CS2d} \cdot \alpha_{2d} + k_{-51} \cdot C_{APPCS2d\alpha2d} \\
\dot{APP}_{E1} &= k_{e1} \cdot APP_{CS1} - k_{-e1} \cdot APP_{E1} - k_3 \cdot APP_{E1} \cdot \beta_1 + k_{-3} \cdot C_{APPE1\beta1} \\
\dot{APP}_{E2d} &= k_{e2} \cdot APP_{CS2d} - k_{-e2} \cdot APP_{E2d} - k_{31} \cdot APP_{E2d} \cdot \beta_{2d} + k_{-31} \cdot C_{APPE2d\beta2d}
\end{aligned}
$$

influence of SORLA

$$
\begin{aligned}
\dot{C}_{APPG1SORLAG1} &= k_{s1} \cdot APP_{G1} \cdot SORLA_{G1} - k_{-s1} \cdot C_{APPG1SORLAG1} \\
\dot{C}_{APPG2SORLAG2} &= k_{s2} \cdot APP_{G2} \cdot SORLA_{G2} - k_{-s2} \cdot C_{APPG2SORLAG2} \\
\dot{SORLA}_{G1} &= -\dot{C}_{APPG1SORLAG1} \\
\dot{SORLA}_{G2} &= -\dot{C}_{APPG2SORLAG2}
\end{aligned}
$$

complex formations of APP and secretases

$$
\begin{aligned}
\dot{C}_{APPCS1\alpha1} &= k_5 \cdot APP_{CS1} \cdot \alpha_1 - (k_{-5} + k_6) \cdot C_{APPCS1\alpha1} \\
\dot{C}_{APPCS2d\alpha2d} &= k_{51} \cdot APP_{CS2d} \cdot \alpha_{2d} - (k_{-51} + k_{61}) \cdot C_{APPCS2d\alpha2d} \\
\dot{C}_{APPE1\beta1} &= k_3 \cdot APP_{E1} \cdot \beta_1 - (k_{-3} + k_4) \cdot C_{APPE1\beta1} \\
\dot{C}_{APPE2d\beta2d} &= k_{31} \cdot APP_{E2d} \cdot \beta_{2d} - (k_{-31} + k_{41}) \cdot C_{APPE2d\beta2d}
\end{aligned}
$$

secretases

$$
\begin{aligned}
\dot{\alpha}_{init} &= k_{-c1} \cdot \alpha_1 + k_{-c2} \cdot \alpha_2 - (k_{c1} + k_{c2}) \cdot \alpha_{init} \\
\dot{\alpha}_1 &= k_{c1} \cdot \alpha_{init} - k_{-c1} \cdot \alpha_1 - \dot{C}_{APPCS1\alpha1} \\
\dot{\alpha}_2 &= k_{c2} \cdot \alpha_{init} - k_{-c2} \cdot \alpha_2 - 2 \cdot (k_{c3} \cdot \alpha_2^2 - k_{-c3} \cdot \alpha_{2d}) \\
\dot{\alpha}_{2d} &= 2 \cdot (k_{c3} \cdot \alpha_2^2 - k_{-c3} \cdot \alpha_{2d}) - \dot{C}_{APPCS2d\alpha2d} \\
\dot{\beta}_{init} &= k_{-b1} \cdot \beta_1 + k_{-b2} \cdot \beta_2 - (k_{b1} + k_{b2}) \cdot \beta_{init} \\
\dot{\beta}_1 &= k_{b1} \cdot \beta_{init} - k_{-b1} \cdot \beta_1 - \dot{C}_{APPE1\beta1} \\
\dot{\beta}_2 &= k_{b2} \cdot \beta_{init} - k_{-b2} \cdot \beta_2 - 2 \cdot (k_{b3} \cdot \beta_2^2 - k_{-b3} \cdot \beta_{2d}) \\
\dot{\beta}_{2d} &= 2 \cdot (k_{b3} \cdot \beta_2^2 - k_{-b3} \cdot \beta_{2d}) - \dot{C}_{APPE2d\beta2d}
\end{aligned}
$$

products

$$
\begin{aligned}
s\dot{APP}\alpha_1 &= k_6 \cdot C_{APPCS1\alpha1} \\
\dot{C83}_1 &= k_6 \cdot C_{APPCS1\alpha1} \\
s\dot{APP}\alpha_2 &= 2 \cdot k_{61} \cdot C_{APPCS2d\alpha2d} \\
\dot{C83}_{2d} &= k_{61} \cdot C_{APPCS2d\alpha2d} \\
s\dot{APP}\beta_1 &= k_4 \cdot C_{APPE1\beta1} \\
\dot{C99}_1 &= k_4 \cdot C_{APPE1\beta1} \\
s\dot{APP}\beta_2 &= 2 \cdot k_{41} \cdot C_{APPE2d\beta2d} \\
\dot{C99}_{2d} &= k_{41} \cdot C_{APPE2d\beta2d}
\end{aligned}
$$

1.3 Assumptions

1.3.1 Quasi-steady state

When quasi-steady state is assumed to a complex, e.g. $C_{APPCS1\alpha1}$, without loss of generality $\dot{C}_{APPCS1\alpha1} = 0$. We will show computational details for this example and the others will follow similarly.

$$
\begin{aligned}
\dot{C}_{APPCS1\alpha1} &= k_5 \cdot APP_{CS1} \cdot \alpha_1 - (k_{-5} + k_6) \cdot C_{APPCS1\alpha1} \\
0 &= k_5 \cdot APP_{CS1} \cdot \alpha_1 - (k_{-5} + k_6) \cdot C_{APPCS1\alpha1} \\
C_{APPCS1\alpha1} &= \frac{k_5 \cdot \alpha_1 \cdot APP_{CS1}}{k_{-5} + k_6}
\end{aligned}
$$

Therefore,

$$
\begin{aligned}
C_{APPCS1\alpha1} &= \frac{\alpha_1 \cdot APP_{CS1}}{K_{M\alpha1}} \quad , \quad K_{M\alpha1} = \frac{k_{-5} + k_6}{k_5} \\
C_{APPE1\beta1} &= \frac{\beta_1 \cdot APP_{E1}}{K_{M\beta1}} \quad , \quad K_{M\beta1} = \frac{k_{-3} + k_4}{k_3} \\
C_{APPCS2d\alpha2d} &= \frac{\alpha_{2d} \cdot APP_{CS2d}}{K_{M\alpha2d}} \quad , \quad K_{M\alpha2d} = \frac{k_{-51} + k_{61}}{k_{51}} \\
C_{APPE2d\beta2d} &= \frac{\beta_{2d} \cdot APP_{E2d}}{K_{M\beta2d}} \quad , \quad K_{M\beta2d} = \frac{k_{-31} + k_{41}}{k_{31}}
\end{aligned}
$$

1.3.2 Rapid-Equilibrium

Without loss of generality, rapid-equilibrium is assumed to $C_{APPG1\dot{S}ORLAG1}$ and $C_{APPG2\dot{S}ORLAG2}$ such that $C_{APPG1\dot{S}ORLAG1} = 0$ and $C_{APPG2\dot{S}ORLAG2} = 0$. The assumption gives rise to,

$$
\begin{aligned}
C_{APPG1SORLAG1} &= K_{S1} \cdot SORLA_{G1} \cdot APP_{G1} \text{ , } where\ K_{S1} = \frac{k_{s1}}{k_{-s1}}, \ and \\
C_{APPG2SORLAG2} &= K_{S2} \cdot SORLA_{G2} \cdot APP_{G2} \text{ , } where\ K_{S2} = \frac{k_{s2}}{k_{-s2}}.
\end{aligned}
$$

1.3.3 Quasi-equilibrium

Recall the biochemical networks of the transportation of reactants shown in previous section,

$$
\begin{array}{lcl}
APP_{init} & \xrightarrow{K_{G1}} & APP_{G1} \\
APP_{init} & \xrightarrow{K_{G2}} & APP_{G2} \\
APP_{G1} & \xrightarrow{K_{CS1}} & APP_{CS1} \\
APP_{G2d} & \xrightarrow{K_{CS2}} & APP_{CS2d} \\
\alpha_{init} & \xrightarrow{K_{C1}} & \alpha_1 \\
\alpha_{init} & \xrightarrow{K_{C2}} & \alpha_2 \\
\beta_{init} & \xrightarrow{K_{B1}} & \beta_1 \\
\beta_{init} & \xrightarrow{K_{B2}} & \beta_2
\end{array}
$$

The second reactant is assumed to be in quasi-equilibrium with the first reactant. Without loss of generality, the concentration of the second reactant is related to the first reactant by an ordinary equilibrium expression, such as

$$
\begin{aligned}
APP_{G1} &= K_{G1} \cdot APP_{init} \ , \ where \ K_{G1} = \frac{k_{g1}}{k_{-g1}}, \\
APP_{G2} &= K_{G2} \cdot APP_{init} \ , \ where \ K_{G2} = \frac{k_{g2}}{k_{-g2}}, \\
APP_{CS1} &= K_{CS1} \cdot APP_{G1} \ , \ where \ K_{CS1} = \frac{k_{cs1}}{k_{-cs1}}, \\
APP_{CS2d} &= K_{CS2} \cdot APP_{G2d} \ , \ where \ K_{CS2} = \frac{k_{cs2}}{k_{-cs2}}, \\
\alpha_1 &= K_{C1} \cdot \alpha_{init} \ , \ where \ K_{C1} = \frac{k_{c1}}{k_{-c1}}, \\
\alpha_2 &= K_{C2} \cdot \alpha_{init} \ , \ where \ K_{C2} = \frac{k_{c2}}{k_{-c2}}, \\
\beta_1 &= K_{B1} \cdot \beta_{init} \ , \ where \ K_{B1} = \frac{k_{b1}}{k_{-b1}}, \\
\beta_2 &= K_{B2} \cdot \beta_{init} \ , \ where \ K_{B2} = \frac{k_{b2}}{k_{-b2}},
\end{aligned}
$$

1.3.4 Ratio of association constant

Recall the biochemical networks of the transportation of reactants shown in previous section,

$$
\begin{array}{lcl}
APP_{CS1} & \underset{k_{-e1}}{\overset{k_{e1}}{\rightleftharpoons}} & APP_{E1} \\
APP_{CS2d} & \underset{k_{-e2}}{\overset{k_{e2}}{\rightleftharpoons}} & APP_{E2d}
\end{array}
$$

and also the dimerization of reactants,

$$
\begin{array}{lclcl}
APP_{G2} & + & APP_{G2} & \underset{k_{-g3}}{\overset{k_{g3}}{\rightleftharpoons}} & APP_{G2d} \\
\alpha_2 & + & \alpha_2 & \underset{k_{-c3}}{\overset{k_{c3}}{\rightleftharpoons}} & \alpha_{2d} \\
\beta_2 & + & \beta_2 & \underset{k_{-b3}}{\overset{k_{b3}}{\rightleftharpoons}} & \beta_{2d}
\end{array}
$$

We take one example and compute for its ratio of association constant:

$$
K_{E1} = \frac{k_{e1}}{k_{-e1}} = \frac{APP_{E1}}{APP_{CS1}}
$$

such that

$$APP_{E1} = K_{E1} \cdot APP_{CS1} \ , \ where \ K_{E1} = \frac{k_{e1}}{k_{-e1}}.$$

The others follow similar approach of computation and are denoted as follows:

$$\begin{aligned}
APP_{E2d} &= K_{E2} \cdot APP_{CS2d} \ , \ where \ K_{E2} = \frac{k_{e2}}{k_{-e2}}, \\
APP_{G2d} &= K_{G3} \cdot APP_{G2}^{2} \ , \ where \ K_{G3} = \frac{k_{g3}}{k_{-g3}}, \\
\alpha_{2d} &= K_{C3} \cdot \alpha_{2}^{2} \ , \ where \ K_{C3} = \frac{k_{c3}}{k_{-c3}}, \\
\beta_{2d} &= K_{B3} \cdot \beta_{2}^{2} \ , \ where \ K_{B3} = \frac{k_{b3}}{k_{-b3}},
\end{aligned}$$

1.3.5 Law of conservation

Law of conservation is assumed to APP, α-secretase, β-secretase, and SORLA. Regardless of SORLA, the total amounts of APP, α-secretase, β-secretase, and SORLA, that are conserved in the system, are represented by the following equations:

$$\begin{aligned}
\alpha_{Tot} &= \alpha_{init} + \alpha_{monomer} + \alpha_{dimer} & (1) \\
\beta_{Tot} &= \beta_{init} + \beta_{monomer} + \beta_{dimer} & (2) \\
APP_{Tot} &= APP_{init} + APP_{monomer} + APP_{dimer} & (3) \\
SORLA_{Tot} &= SORLA_{monomer} + SORLA_{dimer} & (4)
\end{aligned}$$

In order to differentiate the functions in the presence and in the absence of SORLA, the reactants and complexes are denoted as functions with respect to the reactants they depend on in the process of computation. For example, $f(x_1, y_1)$ is the function f that is dependent on the representation of x_1 and y_1.

In the absence of SORLA,

$$\begin{aligned}
\alpha_{monomer} &= \alpha_1(\alpha_{init}) + C_{APPCS1\alpha1}(APP_{init}, \alpha_{init}) \\
\alpha_{dimer} &= \alpha_2(\alpha_{init}) + 2 \cdot [\alpha_{2d}(\alpha_{init}) + C_{APPCS2d\alpha2d}(APP_{init}, \alpha_{init})] \\
\beta_{monomer} &= \beta_1(\beta_{init}) + C_{APPE1\beta1}(APP_{init}, \beta_{init}) \\
\beta_{dimer} &= \beta_2(\beta_{init}) + 2 \cdot [\beta_{2d}(\beta_{init}) + C_{APPE2d\beta2d}(APP_{init}, \beta_{init})] \qquad (5) \\
APP_{monomer} &= APP_{G1}(APP_{init}) + APP_{CS1}(APP_{init}) + APP_{E1}(APP_{init}) + \\
&\quad C_{APPCS1\alpha1}(APP_{init}, \alpha_{init}) + C_{APPE1\beta1}(APP_{init}, \beta_{init}) \\
APP_{dimer} &= APP_{G2}(APP_{init}) + 2 \cdot [APP_{G2d}(APP_{init}) + APP_{CS2d}(APP_{init}) + APP_{E2d}(APP_{init}) + \\
&\quad C_{APPCS2d\alpha2d}(APP_{init}, \alpha_{init}) + C_{APPE2d\beta2d}(APP_{init}, \beta_{init})]
\end{aligned}$$

The equations above are substituted into Equations (1), (2), and (3), APP_{init}, α_{init} and β_{init} are solved with respect to APP_{Tot}, α_{Tot} and β_{Tot}.

For APP_{Tot} without SORLA,

$$\begin{aligned}
APP_{Tot} &= APP_{init} + \\
& K_{G1} \cdot APP_{init} \cdot \left\{1 + K_{CS1} \cdot \left[1 + \frac{K_{C1} \cdot \alpha_{init}}{K_{M\alpha 1}}\right] + K_{E1} \cdot K_{CS1} \cdot \left[1 + \frac{K_{B1} \cdot \beta_{init}}{K_{M\beta 1}}\right]\right\} + \\
& K_{G2} \cdot APP_{init} + 2 \cdot K_{G3} \cdot (K_{G2} \cdot APP_{init})^2 \cdot \left\{1 + K_{CS2} \cdot \left[1 + \frac{K_{C3} \cdot (K_{C2} \cdot \alpha_{init})^2}{K_{M\alpha 2d}}\right] + \right. \\
& \left. K_{E2} \cdot K_{CS2} \cdot \left[1 + \frac{K_{B3} \cdot (K_{B2} \cdot \beta_{init})^2}{K_{M\beta 2d}}\right]\right\} \qquad (6)
\end{aligned}$$

Due to the complexity of Equation (6), the computation of APP_{init} with respect to APP_{Tot} will not be shown algebraically, but it will be solved using fzero() function that is available in MATLAB. Nevertheless, the algebraic computation for α_{init} and β_{init} are shown below. We will show the computation for α_{init} and the solution for β_{init} will follow similarly. Take α_{Tot} shown in Equation (1), i.e.

$$\alpha_{Tot} = \alpha_{init} + \alpha_{monomer} + \alpha_{dimer}$$

$\alpha_{monomer}$ and α_{dimer} from Equation (5) are substituted into Equation (1), which leads to

$$\begin{aligned}
\alpha_{Tot} &= \alpha_{init} + [\alpha_1(\alpha_{init}) + C_{APPCS1\alpha 1}(APP_{init}, \alpha_{init})] + \\
& \{\alpha_2(\alpha_{init}) + 2 \cdot [\alpha_{2d}(\alpha_{init}) + C_{APPCS2d\alpha 2d}(APP_{init}, \alpha_{init})]\}
\end{aligned}$$

Further substitutions of the notations that are derived in the previous sections give

$$\begin{aligned}
\alpha_{Tot} &= \alpha_{init} + K_{C1} \cdot \alpha_{init} \cdot \left(1 + \frac{APP_{CS1}}{K_{M\alpha 1}}\right) + K_{C2} \cdot \alpha_{init} + 2 \cdot K_{C3} \cdot (K_{C2} \cdot \alpha_{init})^2 \cdot \left(1 + \frac{APP_{CS2d}}{K_{M\alpha 2d}}\right) \\
&= \alpha_{init} + K_{C1} \cdot \alpha_{init} \cdot \left(1 + \frac{K_{CS1} \cdot K_{G1} \cdot APP_{init}}{K_{M\alpha 1}}\right) + K_{C2} \cdot \alpha_{init} + \\
& 2 \cdot K_{C3} \cdot (K_{C2} \cdot \alpha_{init})^2 \cdot \left(1 + \frac{K_{CS2} \cdot K_{G3} \cdot (K_{G2} \cdot APP_{init})^2}{K_{M\alpha 2d}}\right) \qquad (7)
\end{aligned}$$

such that

$$\alpha_{init} = \frac{-B_\alpha \pm \sqrt{B_\alpha^2 - 4 \cdot A_\alpha \cdot C_\alpha}}{2 \cdot A_\alpha} \qquad (8)$$

where

$$\begin{aligned}
A_\alpha &= 2 \cdot K_{C3} \cdot K_{C2}^2 \cdot \left(1 + \frac{K_{CS2} \cdot K_{G3} \cdot (K_{G2} \cdot APP_{init})^2}{K_{M\alpha 2d}}\right) \\
B_\alpha &= 1 + K_{C1} \cdot \left(1 + \frac{K_{CS1} \cdot K_{G1} \cdot APP_{init}}{K_{M\alpha 1}}\right) + K_{C2} \\
C_\alpha &= -\alpha_{Tot}.
\end{aligned}$$

Similarly for β_{Tot} shown in Equation (1):

$$\beta_{init} = \frac{-B_\beta \pm \sqrt{B_\beta^2 - 4 \cdot A_\beta \cdot C_\beta}}{2 \cdot A_\beta} \qquad (9)$$

where

$$\begin{aligned}
A_\beta &= 2 \cdot K_{B3} \cdot K_{B2}^2 \cdot \left(1 + \frac{K_{E2} \cdot K_{CS2} \cdot K_{G3} \cdot (K_{G2} \cdot APP_{init})^2}{K_{M\beta 2d}}\right) \\
B_\beta &= 1 + K_{B1} \cdot \left(1 + \frac{K_{E1} \cdot K_{CS1} \cdot K_{G1} \cdot APP_{init}}{K_{M\beta 1}}\right) + K_{B2} \\
C_\beta &= -\beta_{Tot}.
\end{aligned}$$

Note that only the positive solutions are biologically meaningful.

In the presence of SORLA, one of the main objective of our study is to differentiate the influence of SORLA in monomer and in dimer processing. Hence, it is necessary to further assume law of conservation in each processing. Otherwise, the SORLA assigned in each processing will indirectly affect the other processing (which can easily be shown by simple algebraic equations).

Here, we consider SORLA to be directly influencing the amounts of free APP available in monomer and dimer processing without affecting APP_{init}. Again, it is important to emphasize that the reason behind this is because of the objective we set at the beginning of the study. Thus, in the presence of SORLA, the effect of SORLA into the free APP starts with APP_{G1} for monomer processing and APP_{G2} for dimer processing. Henceforth, the equations shown in Equation (5) are rewritten in the following ways:

$$\begin{aligned}
\alpha_{monomer} &= \alpha_1 + C_{APPCS1\alpha 1}(APP_{G1}, \alpha_1) \\
\alpha_{dimer} &= \alpha_2 + 2 \cdot (\alpha_{2d}(\alpha_2) + C_{APPCS2d\alpha 2d}(APP_{G2}, \alpha_2)) \\
\beta_{monomer} &= \beta_1 + C_{APPE1\beta 1}(APP_{G1}, \beta_1) \\
\beta_{dimer} &= \beta_2 + 2 \cdot [\beta_{2d}(\beta_2) + C_{APPE2d\beta 2d}(APP_{G2}, \beta_2)] \\
APP_{monomer} &= APP_{G1} + APP_{CS1}(APP_{G1}) + APP_{E1}(APP_{G1}) + \\
&\quad C_{APPCS1\alpha 1}(APP_{G1}, \alpha_1) + C_{APPE1\beta 1}(APP_{G1}, \beta_1) + \\
&\quad C_{APPG1SORLAG1}(APP_{G1}, SORLA_{G1}) \\
APP_{dimer} &= APP_{G2} + 2 \cdot [APP_{G2d}(APP_{G2}) + APP_{CS2d}(APP_{G2}) + APP_{E2d}(APP_{G2}) + \\
&\quad C_{APPCS2d\alpha 2d}(APP_{G2}, \alpha_2) + C_{APPE2d\beta 2d}(APP_{G2}, \beta_2)] + \\
&\quad C_{APPG2SORLAG2}(APP_{G2}, SORLA_{G2}) \\
SORLA_{monomer} &= SORLA_{G1} + C_{APPG1SORLAG1}(APP_{G1}, SORLA_{G1}) \\
SORLA_{dimer} &= SORLA_{G2} + C_{APPG2SORLAG2}(APP_{G2}, SORLA_{G2})
\end{aligned} \tag{10}$$

Similarly, the equations above are substituted to Equations (1), (2), and (3). APP_{G1}, α_1 and β_1 are solved with respect to the total amount of $APP_{monomer}$, $\alpha_{monomer}$ and $\beta_{monomer}$ concentrations calculated from the case without SORLA. Likewise, APP_{G2}, α_2 and β_2 are solved with respect to the total amount of APP_{dimer}, α_{dimer} and β_{dimer} concentrations derived from the case without SORLA.

Moreover,

$$\begin{aligned}
SORLA_{monomer} &= SORLA_{G1} + C_{APPG1SORLAG1}(APP_{G1}, SORLA_{G1}) \\
&= SORLA_{G1} \cdot (1 + K_{S1} \cdot APP_{G1})
\end{aligned}$$

implies that

$$SORLA_{G1} = SORLA_{monomer} \cdot (1 + K_{S1} \cdot APP_{G1})^{-1}$$

and thus

$$C_{APPG1SORLAG1} = K_{S1} \cdot APP_{G1} \cdot [SORLA_{monomer} \cdot (1 + K_{S1} \cdot APP_{G1})^{-1}]$$

Similarly,

$$C_{APPG2SORLAG2} = K_{S2} \cdot APP_{G2} \cdot [SORLA_{dimer} \cdot (1 + K_{S2} \cdot APP_{G2})^{-1}]$$

For APP_{Tot} with SORLA,

$$\begin{aligned} APP_{Tot} &= APP_{init} + \\ & APP_{G1} \cdot \left\{1 + K_{CS1} \cdot \left[1 + \frac{\alpha_1}{K_{M\alpha 1}}\right] + K_{E1} \cdot K_{CS1} \cdot \left[1 + \frac{\beta_1}{K_{M\beta 1}}\right]\right\} + \\ & K_{S1} \cdot APP_{G1} \cdot \left[\frac{SORLA_{monomer}}{1 + K_{S1} \cdot APP_{G1}}\right] + K_{S2} \cdot APP_{G2} \cdot \left[\frac{SORLA_{dimer}}{1 + K_{S2} \cdot APP_{G2}}\right] + \\ & APP_{G2} + 2 \cdot K_{G3} \cdot APP_{G2}^2 \cdot \left\{1 + K_{CS2} \cdot \left[1 + \frac{K_{C3} \cdot \alpha_2^2}{K_{M\alpha 2d}}\right] + \right. \\ & \left. K_{E2} \cdot K_{CS2} \cdot \left[1 + \frac{K_{B3} \cdot \beta_2^2}{K_{M\beta 2d}}\right]\right\} \end{aligned} \tag{11}$$

Due to the complexity of Equation (11), the computations of APP_{G1} and APP_{G2} will not be shown algebraically; but it will be calculated using fzero() function in MATLAB. Nevertheless, the algebraic computation for α_1, α_2, β_1 and β_2 are shown below. We will show the computation for α_1 and α_2 and the solutions for β_1 and β_2 will follow similarly.

First, we compute the amounts of $\alpha_{monomer}$ and α_{dimer} without SORLA (Equation (5)) given APP_{init} (Equation (6)), α_{init} (Equation (8)) and β_{init} (Equation (9)) that are solved previously, i.e.

$$\begin{aligned} \alpha_{monomer} &= K_{C1} \cdot \alpha_{init} \cdot \left(1 + \frac{K_{CS1} \cdot K_{G1} \cdot APP_{init}}{K_{M\alpha 1}}\right) \\ \alpha_{dimer} &= K_{C2} \cdot \alpha_{init} + 2 \cdot K_{C3} \cdot (K_{C2} \cdot \alpha_{init})^2 \cdot \left(1 + \frac{K_{CS2} \cdot K_{G3} \cdot (K_{G2} \cdot APP_{init})^2}{K_{M\alpha 2d}}\right) \end{aligned}$$

Next, we used the calculated values of $\alpha_{monomer}$ and α_{dimer} (shown right above), and substitute them into Equation 10:

$$\begin{aligned} \alpha_{monomer} &= \alpha_1 + C_{APPCS1\alpha 1}(APP_{G1}, \alpha_1) \\ \alpha_{dimer} &= \alpha_2 + 2 \cdot [\alpha_{2d}(\alpha_2) + C_{APPCS2d\alpha 2d}(APP_{G2}, \alpha_2)] \end{aligned}$$

which can be rewritten as

$$\begin{aligned} \alpha_{monomer} &= \alpha_1 \cdot \left(1 + \frac{K_{CS1} \cdot APP_{G1}}{K_{M\alpha 1}}\right) \\ \alpha_{dimer} &= \alpha_2 + 2 \cdot K_{C3} \cdot \alpha_2^2 \cdot \left(1 + \frac{K_{CS2} \cdot K_{G3} \cdot APP_{G2}^2}{K_{M\alpha 2d}}\right) \end{aligned}$$

Lastly, we solved for α_1 and α_2 with respect to the conserved amount of $\alpha_{monomer}$ and α_{dimer} (calculated previously):

$$\alpha_1 = \alpha_{monomer} \cdot \left(1 + \frac{K_{CS1} \cdot APP_{G1}}{K_{M\alpha 1}}\right)^{-1} \tag{12}$$

$$\alpha_2 = \frac{-1 \pm \sqrt{1 + 8 \cdot \alpha_{dimer} \cdot \left[K_{C3} \cdot \left(1 + \frac{K_{CS2} \cdot K_{G3} \cdot APP_{G2}^2}{K_{M\alpha 2d}}\right)\right]}}{4 \cdot K_{C3} \cdot \left(1 + \frac{K_{CS2} \cdot K_{G3} \cdot APP_{G2}^2}{K_{M\alpha 2d}}\right)} \tag{13}$$

Similarly, take

$$\begin{aligned} \beta_{monomer} &= \beta_1 + C_{APPE1\beta 1}(APP_{G1}, \beta_1) \\ \beta_{dimer} &= \beta_2 + 2 \cdot (\beta_{2d}(\beta_2) + C_{APPE2d\beta 2d}(APP_{G2}, \beta_2)) \end{aligned}$$

such that

$$\beta_1 = \beta_{monomer} \cdot \left(1 + \frac{K_{E1} \cdot K_{CS1} \cdot APP_{G1}}{K_{M\beta 1}}\right)^{-1} \tag{14}$$

$$\beta_2 = \frac{-1 \pm \sqrt{1 + 8 \cdot \beta_{dimer} \cdot \left[K_{B3} \cdot \left(1 + \frac{K_{E2} \cdot K_{CS2} \cdot K_{G3} \cdot APP_{G2}^2}{K_{M\beta 2d}}\right)\right]}}{4 \cdot K_{B3} \cdot \left(1 + \frac{K_{E2} \cdot K_{CS2} \cdot K_{G3} \cdot APP_{G2}^2}{K_{M\beta 2d}}\right)} \tag{15}$$

Note that only the positive solutions are biologically meaningful.

1.4 End products

The representation of the products are summarized below:

For APP-monomer processing,

$$\begin{aligned} sA\dot{P}P\alpha_1 &= k_6 \cdot \frac{\alpha_1 \cdot APP_{CS1}}{K_{M\alpha 1}} \\ sA\dot{P}P\beta_1 &= k_4 \cdot \frac{\beta_1 \cdot APP_{E1}}{K_{M\beta 1}} \end{aligned} \tag{16}$$

while for APP-dimer processing,

$$\begin{aligned} sA\dot{P}P\alpha_2 &= 2 \cdot k_{61} \cdot \frac{\alpha_{2d} \cdot APP_{CS2d}}{K_{M\alpha 2d}} \\ sA\dot{P}P\beta_2 &= 2 \cdot k_{41} \cdot \frac{\beta_{2d} \cdot APP_{E2d}}{K_{M\beta 2d}}. \end{aligned} \tag{17}$$

As a whole, for the compartment model with both APP-monomer and APP-dimer processing,

$$\begin{aligned} sA\dot{P}P\alpha_{Tot} &= sA\dot{P}P\alpha_1 + sA\dot{P}P\alpha_2 \\ sA\dot{P}P\beta_{Tot} &= sA\dot{P}P\beta_1 + sA\dot{P}P\beta_2 \end{aligned} \tag{18}$$

wherein APP_{CS1}, APP_{E1}, APP_{CS2d}, APP_{E2d}, α_1, β_1, α_{2d}, and β_{2d} are functions with respect to APP_{init}, α_{init}, and β_{init} in the absence of SORLA; and they are functions with respect to APP_{G1}, APP_{G2}, α_1, α_2, β_1, β_2, $SORLA_{G1}$, and $SORLA_{G2}$ in the presence of SORLA. All the computations are done with respect to the given or estimated conserved values of APP_{Tot}, α_{Tot}, β_{Tot}, and $SORLA_{Tot}$.

2 List of Additional Tables

Table S1. Description of the variables used in the biochemical network.

Notation	Description
	TGN compartment
APP_{init}	initial APP
APP_{G1}	monomeric form of APP in the monomer processing
APP_{G2}	monomeric form of APP in the dimer processing
APP_{G2d}	dimeric form of APP in the dimer processing
$SORLA_{G1}$	SORLA in the monomer processing
$SORLA_{G2}$	SORLA in the dimer processing
$C_{APPG1SORLAG1}$	complex of APP_{G1} and $SORLA_{G1}$ in the monomer processing
$C_{APPG2SORLAG2}$	complex of APP_{G2} and $SORLA_{G2}$ in the dimer processing
	Cell surface compartment
APP_{CS1}	monomeric form of APP in the monomer processing
APP_{CS2d}	dimeric form of APP in the dimer processing
α_{init}	initial α-secretase
α_1	monomeric form of α-secretase in the monomer processing
α_2	monomeric form of α-secretase in the dimer processing
α_{2d}	dimeric form of α-secretase in the dimer processing
$C_{APPCS1\alpha1}$	complex of APP_{CS1} and α_1, formed within monomer processing
$C_{APPCS2d\alpha2d}$	complex of APP_{CS2d} and α_{2d}, formed within dimer processing
$C83_1$	fragment C83, resulting from monomer processing
$C83_{2d}$	fragment C83-dimer, resulting from dimer processing
$sAPP\alpha_1$	soluble APPα, resulting from monomer processing
$sAPP\alpha_2$	soluble APPα, resulting from dimer processing
	Endosome compartment
APP_{E1}	monomeric form of APP in the monomer processing
APP_{E2d}	dimeric form of APP in the dimer processing
β_{init}	initial β-secretase
β_1	monomeric form of β-secretase in the monomer processing
β_2	monomeric form of β-secretase in the dimer processing
β_{2d}	dimeric form of β-secretase in the dimer processing
$C_{APPE1\beta1}$	complex of APP_{E1} and β_1, formed within monomer processing
$C_{APPE2d\beta2d}$	complex of APP_{E2d} and β_{2d}, formed within dimer processing
$C99_1$	fragment C99 in monomer processing
$C99_{2d}$	fragment C99-dimer in dimer processing
$sAPP\beta_1$	soluble APPβ, resulting from monomer processing
$sAPP\beta_2$	soluble APPβ, resulting from dimer processing

Table S2. Description of the variables and parameters used in the mathematical model.

A. List of variables:

Notation	Unit	Description
APP_{init}	fmol	free initial APP-monomer in TGN
APP_{G1}	fmol	free APP-monomer in monomer processing of TGN
APP_{G2}	fmol	free APP-monomer in dimer processing of TGN
APP_{G2d}	fmol	free APP-dimer in dimer processing of TGN
APP_{CS1}	fmol	free APP-monomer in monomer processing of cell surface
APP_{CS2d}	fmol	free APP-dimer in dimer processing of cell surface
APP_{E1}	fmol	free APP-monomer in monomer processing of endosome
APP_{E2d}	fmol	free APP-dimer in dimer processing of endosome
α_{init}	fmol	free initial α-secretase within cell surface
α_1	fmol	free α-secretase-monomer in monomer processing within cell surface
α_2	fmol	free α-secretase-monomer in dimer processing within cell surface
α_{2d}	fmol	free α-secretase-dimer in dimer processing within cell surface
β_{init}	fmol	free initial β-secretase within endosome
β_1	fmol	free β-secretase-monomer in monomer processing within endosome
β_2	fmol	free β-secretase-monomer in dimer processing within endosome
β_{2d}	fmol	free β-secretase-dimer in dimer processing within endosome
$C_{APPCS1\alpha1}$	fmol	complex of APP_{CS1} and α_1, formed within monomer processing of cell surface
$C_{APPE1\beta1}$	fmol	complex of APP_{E1} and β_1, formed within monomer processing of endosome
$C_{APPCS2d\alpha2d}$	fmol	complex of APP_{CS2d} and α_{2d}, formed within dimer processing of cell surface
$C_{APPE2d\beta2d}$	fmol	complex of APP_{E2d} and β_{2d}, formed within dimer processing of endosome
$sAPP\alpha_1$	fmol	soluble APPα resulting from monomer processing of cell surface
$sAPP\beta_1$	fmol	soluble APPβ resulting from monomer processing of endosome
$sAPP\alpha_2$	fmol	soluble APPα resulting from dimer processing of cell surface
$sAPP\beta_2$	fmol	soluble APPβ resulting from dimer processing of endosome
$SORLA_{G1}$	fmol	free SORLA in the monomer processing of TGN
$SORLA_{G2}$	fmol	free SORLA in the dimer processing of TGN
$C_{APPG1SORLAG1}$	fmol	complex of APP_{G1} and $SORLA_{G1}$ in the monomer processing of TGN
$C_{APPG2SORLAG2}$	fmol	complex of APP_{G2} and $SORLA_{G2}$ in the dimer processing of TGN
$APP_{monomer}$	fmol	total APP conserved in the monomer processing
APP_{dimer}	fmol	total APP conserved in the dimer processing
APP_{Tot}	fmol	total APP conserved in the whole system
$SORLA_{monomer}$	fmol	total SORLA conserved in the monomer processing
$SORLA_{dimer}$	fmol	total SORLA conserved in the dimer processing
$SORLA_{Tot}$	fmol	total SORLA conserved in the whole system
$\alpha_{monomer}$	fmol	total α-secretase conserved in the monomer processing
α_{dimer}	fmol	total α-secretase conserved in the dimer processing
α_{Tot}	fmol	total α-secretase conserved in the whole system
$\beta_{momoner}$	fmol	total β-secretase conserved in the monomer processing
β_{dimer}	fmol	total β-secretase conserved in the dimer processing
β_{Tot}	fmol	total β-secretase conserved in the whole system
$sAPP\alpha_{Tot}$	fmol	total soluble APPα
$sAPP\beta_{Tot}$	fmol	total soluble APPβ

Table S2. Description of the variables and parameters used in the mathematical model.

B. List of parameters:

Notation	Unit	Description
K_{G1}		equilibrium constant of APP_{init} and APP_{G1}
K_{G2}		equilibrium constant of APP_{init} and APP_{G2}
K_{CS1}		equilibrium constant of APP_{G1} and APP_{CS1}
K_{CS2}		equilibrium constant of APP_{G2d} and APP_{CS2d}
K_{E1}		equilibrium constant of APP_{CS1} and APP_{E1}
K_{E2}		equilibrium constant of APP_{CS2d} and APP_{E2d}
K_{C1}		equilibrium constant of α_{init} and α_1
K_{C2}		equilibrium constant of α_{init} and α_2
K_{B1}		equilibrium constant of β_{init} and β_1
K_{B2}		equilibrium constant of β_{init} and β_2
K_{G3}	fmol^{-1}	association constant of APP dimerization
K_{B3}	fmol^{-1}	association constant of β-secretase dimerization
K_{C3}	fmol^{-1}	association constant of α-secretase dimerization
K_{S1}	fmol^{-1}	association constant of APP_{G1} and $SORLA_{G1}$
K_{S2}	fmol^{-1}	association constant of APP_{G2} and $SORLA_{G2}$
k_i	$\text{fmol}^{-1}\cdot \text{h}^{-1}$	binding rate constant (where $i = 1, 3, 5, 31, 51$)
k_j	h^{-1}	dissociation rate constant (where $j = -1, -3, -5, -31, -51, -g3, -b3, -c3, 4, 6, 41, 61$)
k_h	$\text{fmol}^{-1}\cdot \text{h}^{-1}$	dimerization rate constant (where $h = g3, b3, c3$)
k_q	h^{-1}	inflow rate constant (where $q = g1, g2, cs1, cs2, e1, e2, c1, c2, b1, b2$)
k_t	h^{-1}	outflow rate constant (where $q = -g1, -g2, -cs1, -cs2, -e1, -e2, -c1, -c2, -b1, -b2$)
k_r	$\text{fmol}\cdot \text{h}^{-1}$	initial rate (where $r = g0$)
$K_{M\alpha1}$	fmol	defined by $(k_6 + k_{-5})/k_5$
$K_{M\beta1}$	fmol	defined by $(k_4 + k_{-3})/k_3$
$K_{M\alpha2d}$	fmol	defined by $(k_{61} + k_{-51})/k_{51}$
$K_{M\beta2d}$	fmol	defined by $(k_{41} + k_{-31})/k_{31}$

Table S3. Steps in computer simulations.

I Sort experimental data according to the total amount of APP values

II For simulation purpose, the unit of measurements is unified into fmol (i.e. the values of APP_{Tot} is converted from Molar to fmol, wherein the unit of measurement for sAPPα and sAPPβ remains as fmol)

III The initial values of the parameters are randomly assigned using the rand() function.

IV The initial values described in Step III are used by the lsqnonlin() function to estimate the parameter values of the mathematical model, whereby the following tasks are performed:

- i Given the total amount of APP values from the experimental data without SORLA, the fzero()function is used to solve for APP_{init}
- ii Use the APP_{init} that is solved in (i) to solve for α_{init} and β_{init} while simultaneously estimating the values for α_{Tot} and β_{Tot}.
- iii Since, the total amount of APP values measured between the experimental data without SORLA and the experimental data with SORLA are different. Thus, it is necessary to compute for the corresponding free APP in the both cases, i.e. with and without SORLA.
- iv Use the values of APP_{init}, α_{init}, and β_{init} that are calculated in (i) and (ii) to solve for the conserved amount of APP, α-, and β-secretase in monomer and in dimer processing without SORLA, i.e. $APP_{monomer}$, APP_{dimer}, $\alpha_{monomer}$, α_{dimer}, $\beta_{monomer}$, and β_{dimer}.
- v Use $APP_{monomer}$, APP_{dimer}, $\alpha_{monomer}$, α_{dimer}, $\beta_{monomer}$, and β_{dimer} that are solved in (iv) to recalculate for the values of APP_{G1}, APP_{G2}, α_1, α_2, β_1, and β_2, which are influenced by SORLA.
- vi Then, apply APP, α-, and β-secretase that are calculated in (i) and (ii) into the production rate equations of sAPPα and sAPPβ without SORLA, and those in (v) into that of with SORLA.
- vii Lastly, the sum of weighted squares of differences between the experimental data and results in (vi) are being minimized, i.e. the predicted and observed total amount of
  1. sAPPα without SORLA
  2. sAPPβ without SORLA
  3. sAPPα with SORLA
  4. sAPPβ with SORLA
  5. APP with SORLA. (This step is necessary because the free APP in the case of with SORLA are computed indirectly)

Table S4. Estimated parameter values for Figure 2: Residual= 2.15×10^1.

**parameter**	**(units)**	**values**	**parameter**	**(units)**	**values**
α_{Tot}	(fmol)	5.52×10^3	β_{Tot}	(fmol)	1.31×10^1
K_{B3}	(fmol^{-1})	2.55×10^{-2}	K_{C3}	(fmol^{-1})	1.52×10^{-5}
K_{G3}	(fmol^{-1})	4.45×10^{11}			
**Monomer processing**			**Dimer processing**		
K_{G1}		1.19×10^{-9}	K_{G2}		2.00×10^{-7}
K_{CS1}		2.01×10^8	K_{CS2}		2.38×10^0
K_{E1}		7.33×10^{-2}	K_{E2}		2.41×10^0
K_{C1}		1.80×10^1	K_{C2}		1.64×10^3
K_{B1}		2.37×10^1	K_{B2}		1.90×10^2
$SORLA_{monomer}$	(fmol)	1.23×10^1	$SORLA_{dimer}$	(fmol)	2.43×10^5
K_{S1}	(fmol^{-1})	3.16×10^{-9}	K_{S2}	(fmol^{-1})	8.35×10^3
k_6	(h^{-1})	7.29×10^{-4}	k_{61}	(h^{-1})	1.77×10^1
$K_{M\alpha 1}$	(fmol)	1.37×10^{-2}	$K_{M\alpha 2d}$	(fmol)	5.91×10^3
k_4	(h^{-1})	5.25×10^2	k_{41}	(h^{-1})	6.59×10^{-1}
$K_{M\beta 1}$	(fmol)	2.23×10^2	$K_{M\beta 2d}$	(fmol)	6.50×10^1

3 List of Additional Figures

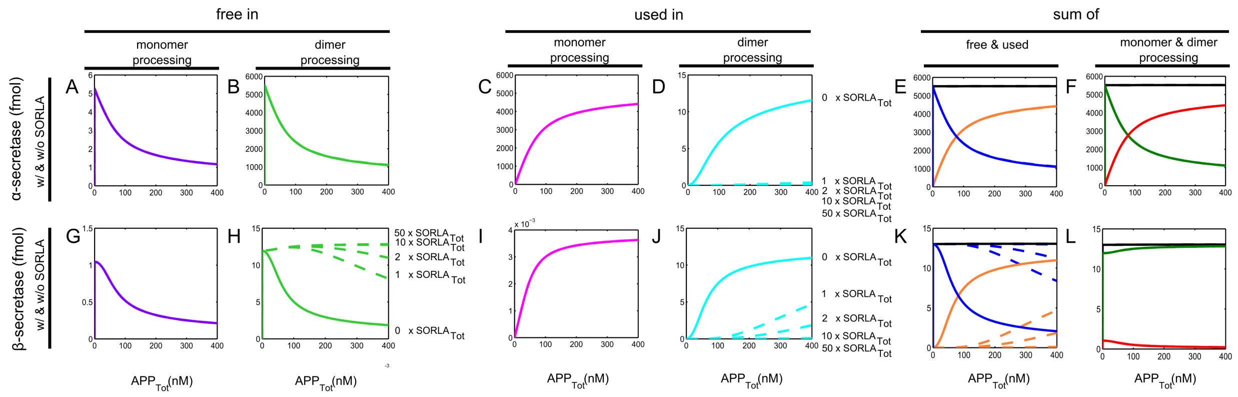

Figure S1: **Concentration values of the secretases with higher SORLA$_{Tot}$ values.** Simulations of the influence of intermediate levels of SORLA on APP processing on the amount of α-secretase (**A-F**) and β-secretase (**G-L**) concentration. The term "used" refers to the complex formation of the secretases and APP, while the term "free" refers to the secretases that are not bound in a complex. There are five intermediate levels of SORLA, namely, 0% (solid line), 100%, 200%, 1000%, and 5000% (dashed line) of SORLA$_{Tot}$ (where SORLA$_{Tot}$ $= 2.43 \times 10^5$ fmol). When there is only solid line in a plot, it is because solid and dashed lines are superimposed. Starting from the first column, there shows the amount of α- (**A**) and β-secretase (**G**) that is free in monomer processing. In the second column, it shows the amount of α- (**B**) and β-secretase (**H**) that is free in dimer processing. The amount of α- (**C**) and β-secretase (**I**) used in monomer processing are shown in the third column, whereas those used in dimer processing (**D, J**) are shown in the fourth column. In the fifth column, there shows the total amount of α- (**E**) and β-secretase (**K**) that is free (blue line) and used (orange line) in the system. Lastly, there is the total amount of α- (**F**) and β-secretase (**L**) in monomer (blue line) and in dimer (orange line) processing of the system. The black lines in (**E, F**) and in (**K, L**) are the estimated total amount of α-and β-secretase, respectively. In particular, the black line in (**E, K**) represents the sum of the secretase concentration depicted by the blue and orange lines, while the one in (**F, L**) indicates the sum of the secretase concentration depicted by the red and green lines. Notice that the solid and dashed lines for both blue and orange colors deviate in (**K**). This, however, is not the case in (**E**).
